# The sea urchin (*Strongylocentrotus purpuratus*) test and spine proteomes

**DOI:** 10.1186/1477-5956-6-22

**Published:** 2008-08-11

**Authors:** Karlheinz Mann, Albert J Poustka, Matthias Mann

**Affiliations:** 1 Max-Planck-Institut für Biochemie, Abteilung Proteomics und Signaltransduktion, D-82152, Martinsried, Am Klopferspitz, 18, Germany; 2 Max-Planck-Institut für Molekulare Genetik, Evolution and Development Group, D-14195, Berlin, Ihnestrasse, 73, Germany

## Abstract

**Background:**

The organic matrix of biominerals plays an important role in biomineral formation and in determining biomineral properties. However, most components of biomineral matrices remain unknown at present. In sea urchin, which is an important model organism for developmental biology and biomineralization, only few matrix components have been identified and characterized at the protein level. The recent publication of the *Strongylocentrotus purpuratus *genome sequence rendered possible not only the identification of possible matrix proteins at the gene level, but also the direct identification of proteins contained in matrices of skeletal elements by in-depth, high-accuracy, proteomic analysis.

**Results:**

We identified 110 proteins as components of sea urchin test and spine organic matrix. Fourty of these proteins occurred in both compartments while others were unique to their respective compartment. More than 95% of the proteins were detected in sea urchin skeletal matrices for the first time. The most abundant protein in both matrices was the previously characterized spicule matrix protein SM50, but at least eight other members of this group, many of them only known as conceptual translation products previously, were identified by mass spectrometric sequence analysis of peptides derived from *in vitro *matrix degradation. The matrices also contained proteins implicated in biomineralization processes previously by inhibition studies using antibodies or specific enzyme inhibitors, such as matrix metalloproteases and members of the mesenchyme-specific MSP130 family. Other components were carbonic anhydrase, collagens, echinonectin, a α2-macroglobulin-like protein and several proteins containing scavenger receptor cysteine-rich domains. A few possible signal transduction pathway components, such as GTP-binding proteins, a semaphorin and a possible tyrosine kinase were also identified.

**Conclusion:**

This report presents the most comprehensive list of sea urchin skeletal matrix proteins available at present. The complex mixture of proteins identified in matrices of the sea urchin skeleton may reflect many different aspects of the mineralization process. Because LC-MS/MS-based methods directly measures peptides our results validate many predicted genes and confirm the existence of the corresponding proteins. Considering the many newly identified matrix proteins, this proteomic study may serve as a road map for the further exploration of biomineralization processes in an important model organism.

## Background

Biominerals contain an organic matrix which is believed to organize a 3-dimensional framework for mineralization, to provide crystal nucleation sites, to contain molecules which determine crystal shape and mineral polymorph, and crystallization inhibitors for termination of crystal growth [1-3]. The sea urchin skeleton consists of calcite rich in magnesium ions and contains less than 0.1% of organic matrix in larval spicules. The skeletal parts analyzed in this report were the shell (test) and the spines of adult animals. The sea urchin test, which protects the internal organs and takes over skeletal functions, consists of small plates which are bound together by an extracellular matrix rich in collagen. The spines, which are used for defense and locomotion, are attached to the test plates by soft tissue at small protuberances called tubercles. The skeletal elements are covered by an epidermis [4]. Both test and spines contain a network of channels and pores, the stereom. The stereom is filled with connective tissue cells, phagocytes, and sclerocytes involved in mineral production and repair [5,6]. Several proteins of the organic matrix were identified by immunological screening of expression libraries, *in situ *hybridization in spicule-forming cells and immunohistochemical methods [7], and their amino acid sequences were predicted from cloned cDNA. However, most of this research concentrated on embryonic spicules [7-10], where skeletogenesis is performed by primary mesenchyme cells (PMCs). The PMCs form syncytia confining the space where biomineralization takes place. During growth, the spicules are enveloped almost completely by a PMC syncytium with plasma membranes in close contact to the extracellular spicule. The PMCs provide the mineral, which is delivered in the form of amorphous calcium carbonate accumulated in intracellular vesicles [11], and the protein precursors for matrix formation. Adult skeletal elements appear to be formed by similar syncytia enclosing a vacuolar cavity containing an organic matrix for mineralization [12]. Amorphous calcium carbonate was also shown to be the mineral precursor in the repair of adult spines [13].

More than 45 spots have been detected by 2-dimensional electrophoresis of the embryonic spicule matrix, but only few of them were identified [14]. Furthermore, only three of the larval spicule matrix proteins, SM50, PM27 and at least one form of SM30, were shown to occur in adult skeletal elements, such as teeth, spines and test, by immunochemical methods [15-17]. Another matrix protein of adult test was carbonic anhydrase [18] which catalyzes the hydration of CO_2 _providing carbonate for mineralization. Recently a genome-wide search for biomineralization-related proteins was performed using the *Strongylocentrotus purpuratus *genome. Genomic sequences were queried with the sequences of known biomineralization-related sequences and the C-type lectin-like domain, which is widespread among sea urchin spicule matrix proteins, to detect related proteins [19]. Special attention was paid to new sequences within genomic clusters of matrix protein genes, genes expressed in biomineral-forming cells at the time of biomineralization, and by screening for sequences similar to vertebrate biomineral matrix proteins. The combined approaches led to the discovery of eight new candidate spicule matrix proteins and several proteins which may not be incorporated into the biomineral but may be involved in the mineralization process. However, the recent completion of the *S. purpuratus *genome [20] also makes possible high-throughput, high-accuracy proteomic analysis of the skeletal matrix and can provide an important complement to genetic studies. In the present report we describe the proteomic analysis of *S. purpuratus *test (shell) and spines by mass spectrometry-based proteomic analysis.

## Methods

### Matrix preparation

Sea urchins were killed by freezing and the tissues were removed mechanically. Three tests were cut into two halves and cleaned by brushing under a jet of de-ionized water. Primary spines were washed by swirling in de-ionized water for approximately 30 min. Further cleaning was done by soaking of tests and spines in 200 ml of sodium hypochlorite solution (NaClO_4; _6–14% active chlorine_; _Merck, Darmstadt, Germany) for 30 min at 4–6°C, with changes after 10 and 20 min, followed by extensive washing with de-ionized water. The dried tests and spines were de-mineralized in 50% acetic acid (20 ml/g of dry biomineral) overnight at 4–6°C. The turbid suspension was dialyzed against 10% acetic acid at 4–6°C, and lyophilized.

### Peptide preparation and data acquisition

**Figure 1:**
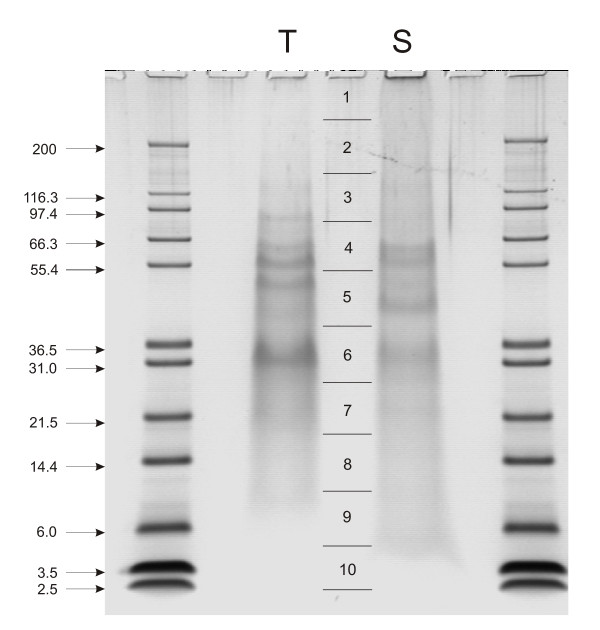
**SDS-PAGE separation of test and spine organic matrix proteins**. The mass of marker proteins is shown in kDa. T, test matrix; S, spine matrix. Approximately 200 μg of matrix were applied per lane. Vertical lines and numbers between lanes T and S indicate the sections excised for in-gel digestion.

SDS-PAGE was done using pre-cast 4–12% Novex Bis-Tris gels in the MES buffer system using reagents and protocols supplied by the manufacturer (Invitrogen, Carlsbad, CA). The kit sample buffer was modified by adding SDS and β-mercaptoethanol to a final concentration of 5%, and the sample was suspended in 40 μl sample buffer/200 μg organic matrix, boiled for 5 min, cooled to room temperature, and centrifuged. Gels were loaded with 200 μg of matrix per lane and stained with colloidal Coomassie (Invitrogen) after electrophoresis. Gel slices were cut out as indicated (Fig. 1) and slices of three lanes were used for in-gel digestion with trypsin [21] in each of three experiments. For analysis of an "empty proteome" sample buffer not containing sea urchin protein was used as input for PAGE. The peptides were cleaned with C18 STAGE-tips before MS analysis [22]. The peptide mixture was separated by nanoscale C18 RP-LC (Agilent 1200; Agilent Technologies, Waldbronn, Germany) coupled on-line to a 7-T LTQ-FT mass spectrometer (Thermo Electron, Bremen, Germany) for LC-MS, MS/MS and MS/MS/MS (MS^3^) analysis [23,24]. 

### Data analysis

Raw data were transformed to msm-files using the in-house-made software RAW2MSM, v.1.10 [25]. The msm-files were used for database searches with the MASCOT search engine (Matrix Science, London, UK; version 2.1) against the *Strongylocentrotus purpuratus *annotated gene models (Glean3) protein sequence database [20] (see also  for further information about Glean [26]), the corresponding reversed database, and the sequences of common contaminants, including human keratins from IPIhuman. Carbamidomethylation was set as fixed modification. Variable modifications were oxidation (M), N-acetyl (protein), pyro (N-term QC), amide (C-terminal) and hydroxylation (P). The peptide tolerance was set to 5 ppm and the MS/MS tolerance was set to 0.5 Da. One miss-cleavage was allowed. MS^3 ^scoring, counting of unique and total peptides, and calculation of protein scores was done with MSQuant, v.1.4.2a13 . Each raw-file was analyzed separately. Msm-files containing data of accepted peptides were then merged into one single msm-file for another Mascot search to obtain summed sequence coverage, scores and peptide numbers. This file was also used to search the IPIhuman database v.3.13 to find shared peptides. The score threshold for peptide acceptance in each Mascot results file was chosen such as to eliminate any reversed hits at p < 0.05 and was between 38 and 54 for different searches. Peptide hits with one unique peptide were accepted only if confirmed by MS^3 ^[23] with a score at least twice the threshold value for MS/MS, and after manual validation. Quality criteria were the assignment of major peaks, occurrence of uninterrupted y- or b-ion series of at least 3 consecutive amino acids, preferred cleavages N-terminal to proline bonds and C-terminal to Asp or Glu bonds, and the presence of a2/b2 ion pairs. The minimal length required for a peptide was seven amino acids.

The abundance of proteins was estimated by calculating the exponentially modified Protein Abundance Index (emPAI) [27] without using retention time data. BLAST analysis was performed with the program provided by NCBI and searching against the non-redundant database for all organisms. FASTA and MPsrch search programs were used as provided by the European Bioinformatics Institute (EBI, ), searching against UniProt Knowledgebase and UniProtKB/Swiss-Prot protein sequence databases. Signal peptides were predicted with SignalP 3.0  and domains were predicted with NCBI Conserved Domain Search [28] and PROSITE [29].

## Results and discussion

Skeletal elements were cleaned with sodium hypochlorite before demineralization. This is an established method to destroy organic material located at the surface of biominerals and leaves intact only intra-crystalline proteins. The yield of organic matrix after demineralization with acetic acid was approximately 8 mg of matrix per g of cleaned, air-dried test and 7.4 mg/g of spines. Part of the matrix was insoluble in 10% acetic acid and precipitated during dialysis. However, we did not separate acid-soluble and acid-insoluble matrix before electrophoresis. Separation of the proteins by PAGE showed a few discrete bands in a background smear of CBB-stainable material (Fig. 1). The gel lanes were cut into 10 slices of approximately equal size for in-gel digestion (Fig. 1) and the eluted peptides were analyzed after extraction and cleaning on a C18 reversed phase. Treatment of all slices was identical and did not depend on staining intensity or stain binding capacity of the separated proteins.

Not counting tentative identifications, we identified 60–70 proteins in each of the matrices. Exact numbers cannot be provided because some proteins appeared to be encoded in several entries while other entries appeared to contain sequences of more than one protein. At least 37 of these proteins occurred in both matrices (See additional file 1: Proteins identified in test and spines). Some additional identifications were classified tentative (See additional file 2: Proteins tentatively identified in test and spine matrix) because they did not meet the stringent criteria applied to identifications shown in the table of Additional file 1 (additional file 1: Proteins identified in test and spines). Tentative identifications were hits with one unique peptide showing high quality spectra and high MASCOT scores, but lacked confirmation by MS^3 ^data. Altogether these numbers were in good agreement with the 40–50 protein spots detected after separation of the larval spicule matrix [14]. We did not observe sequenced peptides passing the applied thresholds and which were not assigned to a protein.

The abundance of proteins was determined by calculating the emPAI [27] which yields a rough estimate of relative concentrations of the proteins. It should, however, not be confounded with exact protein concentration determination which is not yet possible with complex mixtures. Previous experience [30,31] showed that emPAI can be used to classify proteins as major and minor components, but may be misleading for highly modified proteins. However, peptides with modifications such as phosphorylation or glycosylation need specialized techniques of enrichment and detection and were not included in this general survey and therefore did not contribute to emPAI calculation. Selected proteins or protein families will be discussed in the following sections.

### Spicule matrix (SM) proteins and other C-type lectin-like (CLECT) domain-containing proteins

The spicule matrix (SM) proteins characterized by sequence analysis of cDNA clones and ESTs and located in the spicule matrix by immunological techniques share as a common feature the presence of a single, more or less complete, C-type lectin-like (CLECT) domain. The SM proteins can be divided into two groups according to sequence similarities [10,19]. The SM50 subfamily includes SM50, SM37, SM32, SM29, PM (primary mesenchyme cell protein) 27 and three predicted SM29-related proteins. The SM30 subfamily includes SM30 proteins A-F [19]. Another C-type lectin-like presumptive matrix protein, SpC-lectin, apparently does not belong to either of these families [32]. The assignment of most of these proteins as spicule matrix proteins is provisional because only SM50, SM30 and PM27 were shown directly to be matrix proteins by immunological methods. SM50, PM27 and SM30 were also identified immunochemically in adult skeletal elements [15-17]. However, because it is not known whether the antibodies used in these studies would cross-react with translation products of the newly detected genes, we do not know exactly which proteins were detected in previous studies.

Based on MS/MS and MS^3 ^peptide sequence analysis we identified several members of the SM50 subfamily in test or spine matrix (See additional file 1: Proteins identified in test and spines). These were SM50 [Glean3:18811], which was the most abundant protein in both matrices, SM37 [Glean3:18813], SM32 [Glean3:18810], SM29 [Glean3:05990] and three predicted proteins similar to SM29 [Glean3:05989, 05991 and 05992] the genes of which were suggested to code for matrix proteins because of their sequence similarity to SM29 and their position immediately flanking the SM29 gene [19]. Of these, Glean3:05991 was identified in spine matrix only, while Glean3:05992 was only found in test matrix. The SM30 subfamily was represented by SM-30-E and F (See additional file 1: Proteins identified in test and spines), A, B, C and D were absent. SM30-E was highly abundant in spine and test matrix. SM30-F was a low abundance component of spine matrix (See additional file 1: Proteins identified in test and spines) although it was shown previously to be highly expressed at the transcript level in adult spines by RT-PCR [18]. In addition the test matrix contained a protein similar to SM30 [Glean3:27906] which was identified as a candidate matrix protein previously [19]. Interestingly, SM30α (SM30-B [Glean3:00826] according to [19]), which was the first SM30 member characterized by cDNA-derived sequences and found to be among the major proteins of larval spicules [33], was not identified in our proteomic analysis of adult tissues. Its occurrence in adult tissues is controversial. One report [17] identified SM30 by immunoelectron microscopy in the matrix of *S. purpuratus *test while another report using RNA blot analysis failed to detected SM30 message in *H. pulcherrimus *test-related tissues but found traces in spine matrix-related tissues [34]. More recently RNA of SM30-C to -F was found to be expressed in adult spine matrix-producing cells by RT-PCR [19]. However, compared to direct sequencing of matrix proteins from de-mineralized skeletal elements by Edman sequence analysis or MS/MS, immunochemical or RNA-based methods may be considered to produce indirect evidence.

The list of the five most abundant test proteins (See additional file 1: Proteins identified in test and spines) contained two entries with a predicted CLECT domain, Glean3:13825 and Glean3:11163, which did not show sequence similarity to the SM proteins described above. The predicted CLECT domain of 13825 was very rudimentary and did not even contain the highly conserved cysteines commonly found in such domains. However, this domain was followed by a stretch of sequence containing short proline-rich repeats of the type PXX and PXXX, which are common in SM proteins [19]. The predicted CLECT domain of this entry reached from aa23-138 and was most similar to vertebrate lithostathines and chicken mannose receptor C1 (~28% identity). All identified peptides were located in this region. Entry Glean3:11163 contained a much better conserved CLECT domain, but, as in entry Glean3:13825, the highly conserved residues forming the carbohydrate binding site of a typical lectin of this family, such as the nacre protein perlucin [35], were not conserved, indicating that both proteins were not lectins. Entry Glean3:11163 was most similar to mouse macrophage galactose-type CLECT (Swiss-Prot: Q8J2N1) and vertebrate proteoglycan domains such as the brevican CLECT (~30% sequence identity).

We did not find PM27 although it was previously identified by Western blotting in test extract [16]. However, the antiserum raised against PM27 reacted with several bands and it cannot be excluded that the antibodies cross-reacted with other family members. Another reason for its absence could be its localization at the surface of test plates. In spicules at least PM27 was detected only at the surface but not within the mineralized structure [16]. Such surface proteins are likely to be destroyed by NaClO_4 _cleaning.

### Carbonic anhydrase

Carbonic anhydrase catalyzes the reversible hydration of CO_2_, providing carbonate for calcite accumulation. Inhibitors of the enzyme were shown to block spicule formation in the sea urchin embryo [36]. The enzyme activity was also found in adult test [18]. The genome of S. purpuratus contained 19 genes coding for carbonic anhydrases and three of these were expressed at the RNA level in primary mesenchyme cells [19]. RT-PCR studies showed that especially Glean3:012518 RNA was expressed at the time of skeletogenesis, but also in adult spine tissue, indicating that this was the carbonic anhydrase important for skeleton formation and repair [19]. This was confirmed by our identification of this enzyme in test and spine matrix as the only carbonic anhydrase protein detected in both compartments (See additional file 1: Proteins identified in test and spines).

### Collagens

**Figure 2:**
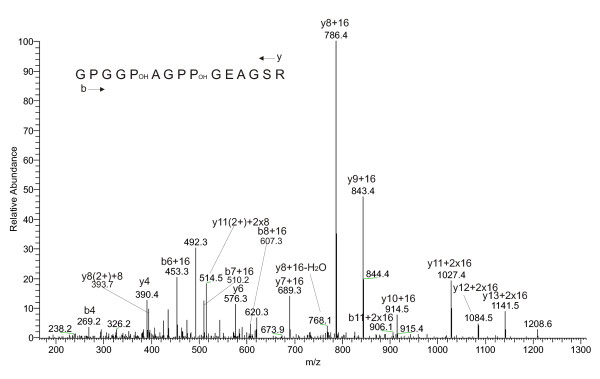
**Mass spectrum of a collagenous peptide with hydroxyproline in X and Y position of the Gly-X-Y triplet**. The MS/MS spectrum showed a y-ion series with an increase of 16 Da starting with y7 and 32 Da starting with y11 indicating hydroxylation of prolines five and nine of the sequence GPGGPAGPPGEAGSR. This was confirmed by a less complete b-ion series.

Previous reports identified several different fibrillar collagen chains in the test of the sea urchins *Asthenosoma ijimai *[37,38] and *Anthocidaris crassispina *[39] using immunochemical and biochemical methods. We identified by MS/MS sequence analysis three Glean3 entries coding for fibrillar collagen chains (Glean3:05167, 26009 and 26008; (See additional file 1: Proteins identified in test and spines). The sequence of entry 05167 encoded the 5α collagen chain of *S. purpuratus *[40] and entries Glean3:26008 and 26009 contained the 1α and 2α collagen chains [41,42]. As expected from collagen maturation pathways all identified peptides matched to the triple-helical domains of the mature collagen chains. Given the known rules of collagen proline hydroxylation and the finding that the number of hydroxyprolines indicated by MASCOT was identical to the number of prolines in Y-position of the Gly-X-Y collagen repeat were identical in 98% of the peptides, we assumed complete hydroxylation of prolines in Y position (See additional file 3: Test and spine collagen peptides containing hydroxyproline). This was confirmed by manual validation of approximately 10% of the 211 spectra of accepted collagen peptides. Manual validation was particularly successful with shorter peptides because in long peptides crucial large fragments were sometimes absent. In addition some peptides (~2%) were found to contain more hydroxyprolines than prolines in Y-position, indicating hydroxylation of some prolines in X-position (See additional file 3: Test and spine collagen peptides containing hydroxyproline, and Fig 2). This is a well characterized collagen modification but is much less frequent than hydroxylation in Y-positions [43].

The collagens were of relatively low abundance (See additional file 1: Proteins identified in test and spines). This was in contrast to the high yields obtained from test of *A. ijimai *[37] and *A. crassispina *[39] where fibrillar collagen was the predominant component. However, these tests were not treated with hypochlorite to clean the crystal surfaces. Most of this collagen was apparently located in sutures between test plates [37]. The collagen identified in the present study was apparently only a small fraction of total test collagen and was protected against hypochlorite-mediated destruction by its location within crystals. Spine intracrystaline matrix contained only small amounts (See additional file 1: Proteins identified in test and spines) of the 1α chain, which could have been part of the spine ligaments connecting spines to test tubercles and were possibly protected by intracrystaline location.

### MSP130 and related proteins

This group of proteins comprises the primary mesenchyme cell-specific membrane-associated glycoprotein MSP130 [44,45] and its close relatives MSP130-related proteins 1 and 2 [32] and MSP130-related-3, -4, -5 and -6 [19]. Antibodies against MSP130 were shown to inhibit Ca^2+ ^accumulation and embryonic skeleton formation [46]. However, subsequent studies showed that the antibodies were directed against a carbohydrate epitope [47] which also occurred on other spicule matrix proteins, such as the matrix protein SM30 [48]. Therefore the function of MSP130 and related proteins remains unclear at present. MSP130 and the related proteins 1–3 and 5 were expressed specifically in PMCs [19,32] while the MSP130-related protein 4 and 6 expression was less clear, leading to the suggestion that the latter proteins were expressed in the adult only or represented pseudogens [19]. None of the MSP130 family members were identified as skeletal matrix proteins previously. We identified five members of the MSP130 family in both, test and spine matrix (See additional file 1: Proteins identified in test and spines). These were MSP130 [Glean3:13821/02088] and the related proteins 1 [Glean3:13822], 2 [Glean3:16506/21385], 3 [Glean3:13823] and 4 [Glean3:14496]. The peptide set identifying MSP130-related-3 extensively overlapped with a peptide set identifying the sequence of entry Glean3:06387 (similar to MSP130). These two entries were treated as one protein. The major difference between these two entries was localized in the N-terminal half were several peptides matched the sequence encoded in entry Glean3:06387 but no peptides were found confirming this stretch of sequence in entry Glean3:13823. In contrast, entry Glean3:13823 contained a short C-terminal region which was missing from entry Glean3:06387 and which was confirmed by two peptide sequences derived from MS/MS spectra (See additional file 4: Analysis of MSP130-related-3 and similar entries). The most abundant member of this family in both matrices was MSP130 (See additional file 1: Proteins identified in test and spines). The MSP130-related proteins 5 and 6 were not found, indicating that their concentration was too low to be detected or that they were absent from spine and test matrix.

### Proteases

Test and spine matrix both contained several proteins similar to matrix metalloproteases (MMP), zinc-containing proteases which degrade extracellular matrix proteins and play a role in matrix protein metabolism, cell migration, apoptosis, membrane fusion and the release and activation of growth factors. Four MMPs were identified in the matrix of test as well as spines (See additional file 1: Proteins identified in test and spines). These were Glean3 entries Glean3:13670, 28749, 28748 and 13669, which corresponded to Sp-MT-MMP-e, Sp-MT-MMP-b, Sp-MT-MMP-h and Sp-MT-MMP-d of [49] (MT, potentially membrane-tethered). Two MMPs were identified in test matrix only. These were entries Glean3:05385 (Sp-MMP-f, [48] and Glean3:12549 Sp-MT-MMP-a, [49]). Another member of this superfamily of metalloproteases, identified in spine matrix only, was found in Glean3:03612, corresponding to astacin metalloprotease 1 [49]. In addition, both matrices contained a hypothetical protein with similarity to zinc carboxypeptidases encoded in Glean3:07682 (See additional file 1: Proteins identified in test and spines) and which was identical to Sp-CPE (carboyxpeptidase E)-like of Angerer et al. [49].

These were the first proteases identified as sea urchin skeletal matrix components. However, MMP inhibitors, such as 1,10-phenanthroline, were shown previously to disrupt spicule formation by primary mesenchyme cells and were proposed to inhibit the formation of the syncytium confining the biomineralization compartment and elongation of existing skeletal elements [50]. Another inhibitor, BB-94 [51,52], was shown not to interfere with syncytium formation and appearance of a first calcite crystal, but to inhibit the elongation of spicules within this compartment. This type of inhibitor was therefore suggested to interfere with the delivery of precipitated calcium carbonate or some mature matrix proteins into the mineralization cavity. While the targets of the inhibited MMPs and their precise functions remain unknown at present, our identification of these proteases in the matrix of test and spines indicated their presence in the mineralization compartment of adult animals and their eventual incorporation into the growing crystals. Considering the known functions of MMPs in extracellular matrix remodeling, we speculate that these proteins take part in the maturation of spine and test matrix proteins.

### Other proteins potentially related to skeletogenesis

Entry Glean3:04746 codes for a hypothetical protein with 79% identity to *P. lividus *fibroblast growth factor receptor-2, which has been implicated in PMC migration and skeleton morphogenesis recently [53]. This protein was identified in test matrix at low abundance (See additional file 1: Proteins identified in test and spines). We did not detect peptides of the tyrosine kinase domain which is contained in Glean3:04747 [54]. This was most probably due to the cytosolic location of this domain.

The spine matrix proteome contained three entries coding for proteins similar to echinonectin ([Glean3:12011, 26843 and 19967]; (See additional file 1: Proteins identified in test and spines). Echinonectin is a *Lytechinus variegatus *homodimeric extracellular matrix protein containing cell attachment and galactose binding sites [55]. Levels of mRNA coding for echinonectin were found to rise in PMCs at the time of syncytium formation and initiation of spicule formation in the sea urchin embryo [56]. Antibodies against *Paracentrotus lividus *PI-nectin, a probable echinonectin homologue [56], were shown to interfere with embryonic skeletogenesis inhibiting patterning and growth of spicules [57]. Both results indicate that echinonectin or related molecules may play a role in skeleton formation in the embryo, for instance by regulating cell-matrix interactions during formation of the syncytia, which provide the compartments in which biomineralization takes place. No role for echinonectin or related molecules in formation or repair of the adult sea urchin skeleton was reported previously and it has not been identified as component of any skeletal element before. Due to the low abundance and consequently low sequence coverage of entries Glean3:26843 and Glean3:19967 it is not clear whether the identified peptides belong to one single echinonectin or several related proteins.

Glean3:18406/18407 was found to code for a protein identified in test and spine matrix (See additional file 1: Proteins identified in test and spines). The gene for this protein is located adjacent to the gene coding for P16 [19], a protein essential for skeletogenesis in the sea urchin embryo [58]. The presence of Gly- and Pro-rich repeats similar to those found in SM proteins and the specific expression of its mRNA in PMCs [19] suggest some role for this protein in spicule mineralization. The relatively high abundance of this protein in our samples at least indicates a function in adult tissue mineralization.

### Miscellaneous proteins

Two entries identified by MS/MS-derived peptide sequences coded for proteins similar to thioester-containing protein ([Glean3:24564 and Glean3:24565]; See additional file 1: Proteins identified in test and spines) and were mapped to the same gene (Gene ID/LOC579544). Glean3:24564 encoded a sequence with two predicted N-terminal α2-macroglobulin-like domains (A2M_N and A2M-N_2) while Glean3:24565 contained two predicted α2-macroglobulin domains (A2M and A2M_2) and the C-terminal α2-macroglobulin receptor-binding domain. Together these entries account for a complete α2-macroglobulin-like protein. Glean3:24565 was identified in spine and test matrix while Glean3:24564 was identified in test matrix only. This may be due to the different abundances of the presumed sea urchin α2-macroglobulin in these skeletal elements.

Both matrices contained many proteins with immunoglobulin (IG) domains (See additional file 1: Proteins identified in test and spines) sometimes accompanied by leucine-rich repeats (LRR; [Glean3:25966 and 05538]) and fibronectin 3 (FN3) domains [Glean3:25966]. Such combinations are typically found in adhesion receptors [59] and these domains may have been shed by cells lining the mineralization compartment.

We also identified several proteins of intracellular location, such as actin, ubiquitin, tubulin and histones. These proteins have amino acid sequences which are highly conserved from unicellular eukaryotes to man, and are widespread in animal tissues and proteomic studies. Some of them are notorious contaminants which are difficult to avoid completely even when the usual precautions are taken. When an msm-file containing the data of all accepted peptides was run against a human database as representative of mammalian databases, 23 proteins shared one or more peptides with human proteins. These entries are shown in italics (See additional file 1: Proteins identified in test and spines). However, only few peptides of only nine of these proteins were detected when we analyzed samples which did not contain added protein (empty proteome). These nine proteins are shown in bold italics (See additional file 1: Proteins identified in test and spines). However, these peptides were found only in gel regions corresponding to sections 1 and 9 (Fig. 1) and the numbers of identified and accepted peptides was lower compared to sea urchin samples. We do not believe that these contaminants contribute substantially to our results, but because we are not aware of a method to uniformly and completely label the spine and test proteins, we cannot determine unequivocally possible contributions from different sources with the available set of identified peptides.

## Conclusion

Using high-throughput, high-accuracy proteomic techniques we have identified more than 100 proteins in the organic matrices of spines and test (See additional file 1: Proteins identified in test and spines; additional file 5: Sequences of unique peptides identified in spine matrix and additional file 6: Sequences of unique peptides identified in test matrix). Many of the identified proteins occurred in both compartments, but we also observed clear qualitative and quantitative differences which may be important for future research. The sea urchin test and spine matrix apparently did not contain only previously known or supposed specific matrix components, but also proteins possibly involved in the regulation of mineral or matrix formation, such as matrix metalloproteases, carboanhydrase, MSP130 family members and fibroblast growth factor receptor-2. We also identified some intracellular proteins (see previous section) and common extracellular matrix components such as collagens. We cannot exclude the possibility that these proteins represent some cellular remnants or extracellular matrix material that may have survived bleaching in stereom cavities, although the stereom is not a closed space and should be accessible to cleaning solutions. The relatively low abundance of collagen, which is a major component of unbleached test and constitutes up to 65% of the total test protein in some sea urchin species [37,38], indicated that the extent of possible contamination by such proteins was limited. Furthermore, the only other biomineral thoroughly analyzed by proteomic methods, the chicken eggshell, is completely devoid of cells but nevertheless contains intracellular proteins, such as endoplasmatic reticulum and Golgi constituents, and extracellular matrix components [30]. Thus it seems possible that these proteins were true components of the intracrystalline matrix and were included into the matrix because they were present in the mineralization compartment when mineral growth was proceeding. Some of these proteins may have reached the mineralization space as by-products of the secretion of specific matrix proteins or of mineral precursors, others were possibly derived from damaged cells of the cell layer confining the mineralization space. We do not believe that their presence in the mineral matrix automatically indicates a function for these proteins in the mineralization process or in the mature biomineral. Rather, the composition of the matrix seemed to reflect the complex processes taking place in and around the mineralization space upon mineral formation and growth. Nevertheless, we hope that the list of sea urchin spine and test matrix proteins, which is the most comprehensive one available at present, may serve as a road map to others interested in biomineralization processes.

## Abbreviations

aa: amino acid; CLECT: C-type lectin (CTL)/C-type lectin-like; MMP: Matrix metalloprotease; PMC: Primary mesenchymal cell; SM: Spicule matrix.

## Competing interests

The authors declare that they have no competing interests.

## Authors' contributions

KM conceived the study, performed organic matrix and peptide isolation and data acquisition. AJP provided the animals. KM and AJP did database searches and annotations. MM supplied methodological expertise. All authors took part in the design of the study and were critically involved in data interpretation and manuscript drafting. All authors read and approved the final manuscript.

## Availability & requirements











## Supplementary Material

Additional file 1 Proteins identified in test and spines. List of proteins identified in the organic matrix of demineralized test plates and spine. Click here for file

Additional file 2 Proteins tentatively identified in test and spine matrix. Identifications with a single unique peptide showing good quality, manually validated, spectra without MS^3 ^confirmation. Click here for file

Additional file 3 Test and spine collagen peptides containing hydroxyproline. List of hydroxyproline-containing collagen peptides and frequency of occurrence. Click here for file

Additional file 4 Analysis of MSP130-related-3 and similar entries. Alignment of amino acid sequences of entries similar to MSP130-related-3. Click here for file

Additional file 5 Sequences of unique peptides identified in spine matrix. List of sequences of accepted peptides from spine matrix. Click here for file

Additional file 6 Sequences of unique peptides identified in test matrix. List of sequences of accepted peptides from test matrix. Click here for file
