# Supplementary material for: The sea urchin (Strongylocentrotus purpuratus) test and spine proteomes
Source: Proteome Sci. 2008 Aug 11;6:22. doi: 10.1186/1477-5956-6-22 (PMC2527298; doi:10.1186/1477-5956-6-22)
Supplement: Additional file 1 — Proteins identified in test and spines. List of proteins identified in the organic matrix of demineralized test plates and spine. [file 1477-5956-6-22-S1.doc]

**Proteins identified in test and spines**

|  |  |  |  |  |  |  |  |  |  |  |
| --- | --- | --- | --- | --- | --- | --- | --- | --- | --- | --- |
| **GLEAN3**  **accession** | **UniProt**  **accession** |  | **Protein** |  | **Protein score** | **Unique pep-tides** | **Total**  **accep-ted**  **pep-tides** | **Sequ-ence**  **cover-age** | **Gel**  **section** | **emPAI** |
|  |  |  |  |  |  |  |  |  |  |  |
| 18811 | P11994 | # | Spicule matrix protein SM50 | **T**  **S** | 1509  1010 | 14  12 | 1187  529 | 61%  51% | 4-8  2-8 | 1968418.4  442.7 |
| 04867 |  |  | Similar to spicule matrix protein SM30/SpSM30-E [19] | **T**  **S** | 949  643 | 10  7 | 397  206 | 36%  21% | 3-8  3-7 | 382.1  45.4 |
| 05990 | Q8MUL0 |  | Spicule matrix protein SM29 | **T**  **S** | 616  485 | 6  5 | 298  112 | 31%  26% | 4-9  5-7,9 | 99.0  58.9 |
| 13825 |  |  | Hypothetical protein; domain: CLECT | **T**  **S** | 655  363 | 6  3 | 162  20 | 21%  14% | 4-9  6,7,9 | 71.0  1.7 |
| 11163 |  |  | Hypothetical protein; domain: CLECT | **T**  **S** | 224  198 | 2  2 | 88  24 | 17%  16% | 5-8  7 | 45.4  9.0 |
| 13821  02088 | P08472 |  | (Similar to) MSP130 | **T**  **S** | 2003  1557 | 17  13 | 488  246 | 30%  21% | 3-8  3-7 | 40.6  27.2 |
| 05989 |  |  | Similar to spicule matrix protein SM29 [19] | **T**  **S** | 525  503 | 6  6 | 109  23 | 37%  36% | 6-9  6,9 | 38.8  4.0 |
| 16506  21385 | Q8MUK7 |  | MSP130-related-2 [32] | **T**  **S** | 3035  630 | 28  6 | 630  78 | 56%  30% | 4-9  4-9 | 36.3  4.2 |
| 18813 | O76450 |  | Spicule matrix protein SM37 | **T**  **S** | 1162  535 | 13  6 | 245  102 | 46%  27% | 4-9  3-8 | 30.6  10.5 |
| 13822 | Q8MUK8 |  | MSP130-related-1 [32] | **T**  **S** | 1040  542 | 11  6 | 178  34 | 27%  16% | 3-7  3-7 | 20.5  3.0 |
| 18810 | Q8MUL1 |  | Spicule matrix protein SM32 | **T**  **S** | 264  419 | 3  5 | 46  193 | 13%  15% | 4-9  4-9 | 20.5  214.4 |
| 12518 |  | # | Similar to carbonic anhydrase (7-like A), domain: CA; expressed in PMCs [19] | **T**  **S** | 665  842 | 7  8 | 67  257 | 12%  16% | 5-9  5-9 | 9.0  52.4 |
| ***00062***  ***02788***  ***00061*** |  |  | ***β-Tubulin (1,2,3), peptide set matches several entries*** | ***T*** | ***1269*** | ***12*** | ***88*** | ***38%*** | ***1-10*** | ***6.8*** |
| 13670 |  |  | Similar to matrix metalloproteinase 14/Sp-MT1-4//Sp-MT-MMP-e [49] | **T**  **S** | 1542  1538 | 15  13 | 218  243 | 28%  24% | 5-9  5-9 | 6.5  7.3 |
| 13823  06387 |  |  | Similar to MSP130; MSP130-related-3 [19]; Similar to MSP130; these entries and the peptide sets identified were extensively overlapping, but not identical (Fig. S2); values calculated for entry 13823 aa419-810 and entry 06387 aa1-597 | **T**  **S** | 2040  1315 | 22  14 | 233  147 | 75%  47% | 3-9  3-9 | 5.9  6.0 |
| ***04721***  ***15276***  ***18079***  ***21496*** |  |  | ***Similar to ubiquitin/polyubiquitin/40S ribosomal S27a fusion protein; all peptides in ubiquitin domain (~aa1-76)*** | ***T***  ***S*** | ***315***  ***200*** | ***3***  ***2*** | ***18***  ***17*** | ***45%***  ***16%*** | ***1-10***  ***1,2,4-6,9*** | ***5.3***  ***1.2*** |
| ***24890***  ***02503*** | ***P08991*** |  | ***Similar to histone H2AV/H2AZ; peptide set overlapping with many similar histone H2A entries*** | ***T***  ***S*** | ***313***  ***200*** | ***3***  ***2*** | ***10***  ***29*** | ***35%***  ***13%*** | ***1-6,8,9***  ***1-9*** | ***4.6***  ***2.2*** |
| 28749 |  |  | Similar to matrix metalloproteinase/MMP10/Sp-MT5.MMP-like 2/Sp-MT-MMP-b [49] | **T**  **S** | 871  790 | 10  8 | 91  88 | 22%  19% | 4-9  5-7,9 | 4.0  2.3 |
| 26008 | Q26634 | # | Fibrillar collagen (1α); triple-helical domain (~aa2371-3520) | **T**  **S** | 1993  349 | 23  5 | 177  9 | 37%  8% | 1-4  1 | 3.6  0.4 |
| 18406  18407 |  |  | Hypothetical protein; Gly-rich; gene close to P16 gene, PMC-specific expression [19] | **T**  **S** | 649  893 | 7  10 | 89  143 | 37%  45% | 4-9  4-9 | 3.6  16.8 |
| 22047 |  |  | Similar to phospholipase A2; domain: PLA2c | **T**  **(S)** | 259  72 | 3  1 | 8  3 | 16%  3% | 6,7  5,7 | 3.6  0.5 |
| 11332 |  |  | Similar to Ca^2+^-activated chloride channel; domains: CLCA_N, vWFA, DUF1973 | **T**  **S** | 1530  1443 | 14  13 | 161  135 | 19%  17% | 5-9  4-9 | 2.2  2.8 |
| 23052 |  |  | Hypothetical protein | **T**  **S** | 187  196 | 2  2 | 4  2 | 15%  15% | 6,7  7 | 2.2  1.2 |
| ***00685*** |  |  | ***Similar to histone H4/Sp-early histone H4; peptide set matches numerous similar histone entries*** | ***T***  ***S*** | ***323***  ***392*** | ***3***  ***4*** | ***14***  ***46*** | ***29%***  ***40%*** | ***6-9***  ***1,3,5-9*** | ***2.2***  ***3.6*** |
| 27169 |  |  | Similar to fibulin-6; domains: 1 IGcam, 1 IGc2; no similarity to fibulin observed! | **T**  **S** | 605  804 | 6  8 | 32  25 | 25%  33% | 4-8  4-8 | 1.9  3.0 |
| 26997 |  |  | Hypothetical protein, partial | **T** | 258 | 3 | 7 | 17% | 4 | 1.7 |
| 05167 | Q26637 | # | Similar to α5 collagen (5α); triple-helical domain (~aa1411-2425) | **T** | 1501 | 17 | 114 | 28% | 1-4,8 | 1.5 |
| *05032* |  |  | *Similar to calmodulin 2; peptides match to numerous similar entries* | ***T*** | *192* | *2* | *9* |  | *2,4-10* | *1.5* |
| 00469 |  |  | Similar to novel hemicentin protein (aa276-571)/similar to OCAM (aa362-571); domains: 5 complete, 1 partial IG/IGcam | **T** | 803 | 7 | 35 | 19% | 3-8 | 1.5 |
| *00379* |  |  | *Similar to ribosomal protein L23a* | ***T*** | *254* | *2* | *4* | *14%* | *1,2* | *1.5* |
| 22366 |  |  | Similar to intrinsic factor-B12 receptor; domains: 5 CUB | **T**  **S** | 1359  301 | 15  3 | 111  9 | 18%  3% | 1-8  6 | 1.4  0.2 |
| 27906 |  |  | Hypothetical protein/SpC-lectin PMC1 (SM30-like [19]) | **T**  **S** | 258  262 | 3  3 | 37  22 | 10%  10% | 3-9  4,5 | 1.2  1.2 |
| ***03825*** |  |  | ***Similar to 14-3-3 protein homolog isoform 1/2/Sp-14-3-3 epsilon*** | ***T*** | ***350*** | ***4*** | ***26*** | ***12%*** | ***1-3,5-10*** | ***1.1*** |
| 25966 |  |  | Hypothetical protein/LRR/Ig receptor/Sp-LRR15-like; domains: Leucine-rich repeat; IG, fibronectin (FN)3 | **T**  **S** | 1026  760 | 11  8 | 50  31 | 12%  10% | 3-9  3-9 | 0.9  0.6 |
| 10169 |  |  | Hypothetical protein; domains: 2 IG (immunoglobulin)_like | **T** | 404 | 5 | 20 | 18% | 4-9 | 0.8 |
| 26949 |  |  | Similar to melanotransferrin/EOS47/Sp-Tf; domains: 2 Transferrin (~aa-703-1392); all identified peptides in these domains | **T**  **S** | 922  1811 | 8  15 | 35  111 | 16%  29% | 3-8  2-6 | 0.8  1.3 |
| 05385 |  |  | Similar to membrane-type MMP-1α/Sp-MT5.MMP-like 3/Sp-MMP-f [49] | **T** | 506 | 5 | 24 | 15% | 4-9 | 0.8 |
| *13819*  *13820* |  |  | *Hypothetical protein/Sp-Eno 1/3/α/β-enolase; domain: enolase* | ***T*** | *222* | *2* | *27* | *8%* | *1-10* | *0.7* |
| 20457 |  |  | Hypothetical protein/Sp-B7-like 3; domains: 2 IG | **T** | 253 | 3 | 4 | 8% | 4,6 | 0.7 |
| 05992 |  |  | Similar to matrix protein SM29 (aa1-130) [19] | **T** | 306 | 3 | 23 | 4% | 4-8 | 0.7 |
| ***00595*** | ***Q6PTG0*** |  | ***Elongation factor 1α*** | ***T*** | ***254*** | ***3*** | ***22*** | ***14%*** | ***1-10*** | ***0.6*** |
| ***09477***  ***09165*** |  |  | ***Similar to HSP70/Sp-HSP701C;***  ***Similar to 71kDa cognate HSP/Sp-HSP701A***  ***(overlapping peptide sets for two entries)*** | ***T*** | ***662*** | ***7*** | ***37*** | ***10%*** | ***1-5,7-10*** | ***0.6*** |
| *14869*  *06211* |  |  | *Similar to thioredoxin peroxidase/peroxiredoxin; domain: PRX_Typ2cys* | ***T*** | *250* | *3* | *18* | *9%* | *1-3,5-10* | *0.6* |
| 25068 |  |  | Similar to tetraspanin | **T** | 141 | 1 | 6 | 4% | 1-3,7 | 0.6 |
| 07682 |  |  | Hypothetical protein/Sp-carboxypeptidaseE-like; domain: Peptidase_M14 (Zinc carboxypeptidase) | **T**  **S** | 316  212 | 3  3 | 14  5 | 8%  8% | 4-9  5,6 | 0.6  0.4 |
| 12549 |  |  | Similar to MMP-14/Sp-MT1-4.MMP-like 4/Sp-MT-MMP-a [49] | **T** | 370 | 4 | 5 | 8% | 6,7 | 0.6 |
| 24565  24564 |  |  | Similar to thioester-containing protein/a2-macroglobulin/Sp-cD109-like; domains: A2M, A2M_2, A2M_receptor-binding (24565); A2M_N, A2M-N-2 (24564); | **T**  **S** | 1000  189 | 9  2 | 29  4 | 8%  2% | 2-6  5,6 | 0.5  0.1 |
| *20322*  *24082* |  |  | *Similar to HSP90, partial/Sp-HSP902A1; domains: HATPase_c (aa40-194), partial HSP90 (N-term.)*  *Hypothetical protein; domain: HSP90 (C-term.); possibly C-terminus of Glean3_20322* | ***T***  ***(T)***  ***(S)*** | *460*  *60*  *47* | *4*  *1*  *1* | *29*  *7*  *1* | *9%*  *3%*  *3%* | *1-10*  *1-10*  *1* | *0.5*  *0.2*  *0.2* |
| 26009 | Q26639 | # | α2 collagen (2α); triple-helical domain: ~aa | **T** | 607 | 7 | 29 | 11% | 1-4 | 0.5 |
| 28748 | Q462F5 |  | Matrix metalloprotease (MMP) 1/Sp-MT1-4.MMP-like 7/Sp-MT-MMP-h [49] | **T**  **S** | 495  180 | 5  2 | 18  6 | 12%  3% | 4-8  5,6 | 0.5  0.2 |
| 21260 |  |  | Hypothetical protein | **T**  **S** | 200  191 | 2  2 | 5  5 | 6%  6% | 5  5 | 0.5  0.5 |
| 26000 |  |  | Hypothetical protein/adhesion receptor; domain: IG | **T**  **S** | 531  360 | 6  4 | 15  8 | 11%  6% | 5-7  5,6 | 0.5  0.3 |
| 11180 |  |  | Hypothetical protein/adhesion receptor; domains: EGF-Ca, FN3 | **T**  **S** | 257  181 | 4  2 | 7  6 | 5%  3% | 4,5  4,5 | 0.5  0.2 |
| 04746 |  |  | Hypothetical protein;79% indentity to *P. lividus* FGF-receptor 2 [53]; domains: 1 IG, 2 IGcam | **T** | 206 | 3 | 8 | 9% | 5-7 | 0.5 |
| *23217* |  |  | *Similar to GTP-binding nuclear protein RAN; domain: Ran;* | ***T*** | *178* | *2* | *5* | *9%* | *1-4,10* | *0.5* |
| *08560* |  |  | *Hypothetical protein/Sp-HSP702A; domain: HSP70* | ***T*** | *428* | *4* | *15* | *9%* | *1-10* | *0.4* |
| 01892 |  |  | Hypothetical protein, partial; domain: partial semaphorin | **T** | 192 | 2 | 3 | 5% | 3,4 | 0.4 |
| 00439 |  |  | Hypothetical protein isoform 2 | **T**  **(S)** | 304  68 | 3  1 | 10  1 | 7%  2% | 4,5,8  4 | 0.4  0.1 |
| 05238 |  |  | Hypothetical protein; domain: PSI (occurring in Plexins, Semaphorins and Integrins | **T** | 240 | 2 | 4 | 6% | 4,5 | 0.4 |
| 25926  25927 |  |  | Similar to CG18405-PB; domain: partial semaphorin (N-terminal)  Similar to CG18405-PB; domain: partial semaphorin (C-terminal) | **T** | 265 | 3 | 4 | 10% | 3,4,6 | 0.4 |
| 25235 |  |  | Similar to Egfl6-prov protein; domains: EGF_Ca; CCP (SUSHI repeat), 2 partial vWA_Matrilin; | **T**  **S** | 422  223 | 4  2 | 11  5 | 3%  2% | 4-9  7,8 | 0.3  0.2 |
| 13669 |  |  | Similar to MMP/Sp-MZ1-4.MMP-like 5/Sp-MT-MMP-d [49] | **T**  **S** | 206  370 | 2  4 | 9  11 | 3%  12% | 5-9  5-9 | 0.3  1.4 |
| *24103* |  |  | *Similar to mitochondrial HSP56/Sp-HSPD1* | ***T*** | *285* | *3* | *11* | *4%* | *1,4-6,9,10* | *0.2* |
| 13301 | P19615 |  | major yolk protein (vitellogenin)/Sp-MYP | **T**  **(S)** | 570  58 | 6  1 | 13  1 | 4%  <1% | 2-4  2 | 0.2  <0.1 |
| 05538 |  |  | Hypothetical protein/Sp-LRR/Ig receptor; domains: partial leucine-rich repeat, IGcam | **T**  **S** | 161  240 | 2  3 | 3  5 | 4%  5% | 7,8  7,8 | 0.2  0.3 |
| 14496 |  |  | MSP130-related protein-4 [19] | **T**  **S** | 191  183 | 2  2 | 2  2 | 3%  3% | 4  3 | 0.2  0.2 |
| 00438 |  |  | Similar to peptidylaminoacyl L/D-isomerase | **T** | 101 | 1 | 1 | 2% | 4 | 0.2 |
| *00552*  *03162*  *03496*  *26944* |  |  | *Similar to ADP-ribosylation factor 1/Sp-Arf1/Sp-Arf1L1/Sp-Arf1L1/Sp-Arf4/5* | ***S*** | *105* | *1* | *4* | *6%* | *6,7,9* | *0.2* |
| 22631 |  |  | Similar to TRIAD 1 type I | **T** | 88 | 1 | 2 | 2% | 5,6 | 0.2 |
| *27527* | *Q868Z7* |  | *HSP gp96/Sp-HSP901; domains: HATPase_c, HSP90* | ***T*** | *274* | *2* | *17* | *3%* | *1-7,9,10* | *0.1* |
| 26629  26630  21630 |  |  | Similar to fibulin(-1) | **T** | 205 | 3 | 6 | 2% | 3 | 0.1 |
| 05014 |  |  | Hypothetical protein; domains: scavenger receptor Cys-rich (SR), WSC (putative carbohydrate-binding) | **T** | 125 | 1 | 1 | 1% | 3 | 0.1 |
| 23115 |  |  | Similar to brain RPTPmam4 isoform II/Sp-PTPRiz; domains: 12 fibronectin (FN3), 2 protein tyrosine phosphatase (PTPc) | **T**  **S** | 453  178 | 6  2 | 14  4 | 3%  <1% | 3,4,6  3,6 | 0.1  <0.1 |
| 27145 |  |  | Similar to Cek8/similar to ephedrine receptor/Sp-ephrin; domains: Tyrosine kinase, FN3, SAM, Ephrin_lbd | **T**  **S** | 187  116 | 2  1 | 4  1 | 2%  1% | 4,5  5 | 0.1  <0.1 |
| 06812 |  |  | Hypothetical protein; domains: 1 IG, 2 IGcam | **T** | 167 | 2 | 3 | 2% | 5 | 0.1 |
| 28450 |  |  | Hypothetical protein; domains: 2 IG | **T** | 207 | 2 | 3 | 3% | 6-8 | 0.1 |
| 01796 |  |  | Similar to *L. variegatus* embryonic blastocoelar extracellular matrix protein (FREM2 homolog) | **S**  **(T)** | 553  83 | 5  1 | 15  1 | 4%  <1% | 1-3  3 | 0.3  <0.1 |
| ***09481***  ***09482***  ***09483*** |  |  | ***Actin/Sp-cytoskeletal actin Ia, IIa, IIb*** | ***S*** | ***926*** | ***10*** | ***116*** | ***33%*** | ***1,3-7,9*** | ***4.8*** |
| ***16845***  ***16846***  ***19138***  ***23661***  ***24343*** |  |  | ***Similar to late histone H2B.L4/H2B-1/Sp-late histone H2bh/H2bi/H2be/H2ba/H2bg*** | ***S*** | ***319*** | ***3*** | ***25*** | ***28%*** | ***1-7*** | ***4.2*** |
| 24730  04876 |  |  | Similar to ferritin/Sp-Fth1 | **S** | 359 | 4 | 87 | 23% | 1-3,5-9 | 4.0 |
| 12011 |  |  | Similar to echinonectin; domains: 2 FA58C/discoidin | **S** | 476 | 5 | 50 | 21% | 1,3 | 3.0 |
| 05991 |  |  | Similar to SM29 [19] | **S**  **(T)** | 379 | 6  1 | 18  2 | 34%  5% | 6,7  6,7 | 2.6  0.3 |
| 11106 |  |  | Similar to annexin A4; domains: 4 annexin | **S** | 907 | 9 | 51 | 29% | 1-3,5,6 | 1.7 |
| 09549 |  |  | Similar to Sdcbp-prov protein; N-terminus acetylated (Ser2); domains: 2 PDZ | **S**  **(T)** | 355 | 4  1 | 21  3 | 15%  3% | 1,2,5  5,6 | 1.2  0.9 |
| 28135 | P15870 |  | Histone H1-δ/Sp-H1-0; N-term: N-acetyl-Ala2 | **S** | 138 | 2 | 2 | 12% | 1 | 1.1 |
| 27236 |  |  | Similar to voltage-dependent anion channel 2; domain: Porin_3; N-terminus: acetylated Ala2 | **S** | 388 | 4 | 16 | 16% | 5,6 | 0.8 |
| 23016 |  |  | Similar to *L.variegatus* extracellular matrix protein 3 (~89% identity)/Sp-FREM1-like | **S** | 993 | 10 | 44 | 8% | 2-6 | 0.8 |
| 03918 |  |  | Similar to vitellogenin receptor; domains: 1 EGF_Ca, 4 LY | **S**  **(T)** | 496  100 | 4  1 | 9  1 | 12%  3% | 3,4  4 | 0.8  0.2 |
| 07231 |  |  | similar to tetraspanin family protein | **S** | 230 | 2 | 28 | 5% | 3-5 | 0.6 |
| *14564*  *14567*  *14568*  *22298* |  |  | *Hypothetical protein/Sp-stom; domain: Band_7_stomatin_like* | ***S*** | *233* | *3* | *8* | *10%* | *6* | *0.6* |
| 04869 |  |  | Similar to 30kDa spicule matrix protein/SM30-F [19] | **S** | 187 | 1 | 4 | 6% | 4,5 | 0.5 |
| 04105 |  |  | Similar to T cell-specific protein | **S** | 141 | 2 | 3 | 9% | 6 | 0.5 |
| 14715 |  |  | Similar to gelsolin | **S** | 154 | 2 | 4 | 11% | 1,2,4,5 | 0.5 |
| 24352 |  |  | Similar to ENSANGP00000017306/Sp-mucin-like; domains: NIDO, AMOP, VWD | **S** | 341 | 4 | 20 | 3% | 4,7,8 | 0.4 |
| 26843 |  |  | Similar to echinonectin, partial; domains: 2 FA58C/discoidin | **S** | 288 | 3 | 3 | 9% | 3 | 0.4 |
| 27372 | O97378 |  | Scavenger receptor Cys-rich protein | **S** | 189 | 2 | 13 | 2% | 3 | 0.3 |
| *12112* |  |  | *Similar to Rab 5/Sp-Rab5L* | ***S*** | *121* | *1* | *2* | *5%* | *6* | *0.3* |
| 22561 |  |  | Hypothetical protein/Sp-multiple inositol polyphosphate histidine phosphatase 1; domain: Acid_phosphatase_A | **S** | 143 | 2 | 3 | 5% | 3,4,6 | 0.3 |
| 19967 |  |  | Similar to echinonectin, partial; domains: 4 FA58C/discoidin | **S** | 232 | 3 | 10 | 4% | 2,3 | 0.2 |
| *00503*  *00932* |  |  | *Similar to Rab11b* | ***S*** | *93* | *1* | *1* | *5%* | *6* | *0.2* |
| 08305 |  |  | Similar to cyclophilin/Sp-Peptidylprolyl isomerase (cyclophilin)-like-7; domain: cyclophilin_ABH_like; expressed at low levels in PMCs [19] | **S** | 97 | 1 | 2 | 4% | 7,9 | 0.2 |
| 11588 |  |  | Similar to fibrillar collagen; ~25% identity to α2 and α5 N-propeptide; domains: KAZAL, ZIP (Zinc transporter protein). | **S** | 373 | 5 | 7 | 1% | 2,3 | 0.2 |
| 00513 |  |  | Similar to dual oxidase 1; domains: Animal_hem_  peroxidase, EF_hand, Ferric_reductase_  transmembrane FAD_binding, NAD_binding | **S** | 408 | 5 | 6 | 2% | 1,2 | 0.2 |
| 19691 |  |  | Similar to ENSANGP00000009431; domain: Band_7_flotillin | **S** | 137 | 2 | 3 | 4% | 6,7,9 | 0.2 |
| 03612 |  |  | Hypothetical protein; domain: ZnMc_astacin-like;  Astacin metalloproteinase 1 of [49] | **S** | 121 | 1 | 2 | 3% | 6 | 0.1 |
| 05872  26431 |  |  | Similar to plexin A2/Sp-Sema/PSI | **S** | 127 | 1 | 1 | 1% | 3 | 0.1 |

Proteins are ordered according to decreasing emPAI in test. At the end of the table proteins occurring in spines only are listed according to their emPAI. The average absolute mass accuracy was 0.6ppm for test proteins and 1.4ppm for spine proteins (p<0.05). Protein scores were calculated with MSQuant from unique peptide scores including MS^3^ scores. If the protein was identified in more than three gel sections only sections containing more than 5% of the total peptide number are indicated. #, identified in test and spine matrix previously by biochemical or immunological methods. T, test (shell); S, spines; brackets indicate tentative identification in the respective compartment. Sequence coverage and emPAI were calculated for mature proteins. Unknown signal peptides were predicted (SignalP 3.0; [www.cbs.dtu.dk/services/SignalP](http://www.cbs.dtu.dk/services/SignalP)). The sequences of unique peptides identified are given in Additional file 5: Sequences of unique peptides identified in spine matrix, and Additional file 6: Sequences of unique peptides identified in test matrix. Italics indicate proteins which share identified and accepted peptides with human proteins. Bold italics indicate results which may include some contributions from different sources (see Results and Discussion section, ***Miscellaneous proteins***, of the article. For references see reference list of the article. The sequences of unique peptides are shown in additional files 5 and 6 (See additional file 5: Sequences of unique peptides identified in spine matrix, and additional file 6: Sequences of unique peptides identified in test matrix).
