# Supplementary material for: The sea urchin (Strongylocentrotus purpuratus) test and spine proteomes
Source: Proteome Sci. 2008 Aug 11;6:22. doi: 10.1186/1477-5956-6-22 (PMC2527298; doi:10.1186/1477-5956-6-22)
Supplement: Additional file 2 — Proteins tentatively identified in test and spine matrix. Identifications with a single unique peptide showing good quality, manually validated, spectra without MS3 confirmation. [file 1477-5956-6-22-S2.doc]

**Proteins tentatively identified in test and spine matrix**

| **GLEAN3**  **accession** | **UniProt**  **accession** |  | **Protein** |  | **Peptide score** | **Unique peptides** | **Accep-ted**  **peptides** | **Sequence**  **cover-age** | **Gel**  **section** | **emPAI** |
| --- | --- | --- | --- | --- | --- | --- | --- | --- | --- | --- |
|  |  |  |  |  |  |  |  |  |  |  |
| 09352 |  |  | Hypothetical protein; ~28% identity to sea squirt (*Ciona intestinalis*) globin aa15-129 | **T**  **S** | 69  69 | 1  1 | 2  7 | 8%  8% | 8  6-8 | 2.2  2.2 |
| 09874 |  |  | Hypothetical protein; Gln-rich | **T** | 80 | 1 | 1 | 9% | 4 | 0.8 |
| 13736 |  |  | Hypothetical protein; ~37%identity to S. purpuratus liver fatty acid-binding protein-2 and cellular retinol-binding protein 1b | **T** | 79 | 1 | 2 | 12% | 8 | 0.5 |
| 17985 |  |  | Similar to fibropellin Ia; domain: avidin_2 | **T**  **S** | 68  76 | 1  1 | 4  4 | 4%  4% | 6-8  6-8 | 0.3  0.3 |
| 11256 |  |  | Similar to phospholipase A2; domain: PLA2c | **T**  **S** | 52  53 | 1  1 | 5  3 | 5%  5% | 6-8  7,8 | 0.3  0.3 |
| 06172 |  |  | Similar to related to cofilin; domain: ADF (actin-depolymerizing factor) | **T**  **S** | 83  53 | 1  1 | 1  1 | 7%  7% | 7  7 | 0.3  0.3 |
| 14207 |  |  | Hypothetical protein; domain: partial semaphorin | **T**  **S** | 60  72 | 1  1 | 2  3 | 5%  5% | 3,4  3,4 | 0.3  0.3 |
| 07221 |  |  | Similar to ceruloplasmin; domain: partial Cu-oxidase | **T**  **S** | 92  73 | 1  1 | 1  1 | 2%  2% | 3  3 | 0.2  0.2 |
| 00750 |  |  | Hypothetical protein | **T**  **S** | 98  74 | 1  1 | 1  2 | 30%  30% | 8  8 | 0.2  0.2 |
| 12486 |  |  | Similar to MEGF11; domain: EMI (N-terminal Cys-rich EMILIN | **T** | 57 | 1 | 2 | 4% | 6,7 | 0.2 |
| 09875 |  |  | Hypothetical protein; domains: 4 KAZAL proteinase inhibitor | **T** | 76 | 1 | 1 | 3% | 4 | 0.2 |
| 13140 |  |  | Similar to met proto-oncogene precursor/Sp-RON; domains: semaphorin, 3 plexin repeats, Tyr kinase | **T** | 47 | 1 | 2 | <1% | 3 | 0.1 |
| 04758 |  |  | Similar to egg bindin receptor 1; domains: 3 Hyalin repeats, 2 fibronectin (FN)3 | **T** | 61 | 1 | 1 | 1% | 4 | 0.1 |
| 26637  00377  05658 |  |  | Similar to GRAAL 2, probably N-terminal  Similar to GRAAL 2, probably C-terminal | **T**  **S** | 107  50 | 1  1 | 1  1 | 1%  5% | 3  7 | 0.1  0.3but |
| 20031 |  |  | Similar to peptidyl-glycine α-amidating monooxygenase-B; domain: Cu2_monoox_C | **T** | 59 | 1 | 1 | <1% | 5 | 0.1 |
| 21793  26183 |  |  | Hypothetical protein/Sp-Slc8a2; domains: 2 Na_Ca_exchanger, 2 Calx_beta | **T** | 54 | 1 | 1 | 1% | 6 | 0.1 |
| 23410  01888 |  |  | Similar to complement-related long protein; domains: 2 complement control protein (CCP)/sushi repeat | **T** | 58 | 1 | 2 | 1% | 3 | 0.1 |
| 00752 |  |  | Hypothetical protein | **T** | 114 | 1 | 1 | 1% | 4 | 0.1 |
| 03084 | A0SVR2 |  | Glypican-6 | **T** | 56 | 1 | 1 | 1% | 5 | 0.1 |
| 19695  25836 |  |  | Hypothetical protein/Sp-IGF2R; domains: 9 complete and 2 partial CIMR (cation-independent mannose-6-phosphate receptor repeat) | **T** | 64 | 1 | 5 | <1% | 2,3 | <0.1 |
| 14617  16179  23953 |  |  | Similar to LDL receptor-related 4; domains: 1 CUB, 4 LY, 1 CCP, 1 IG | **T** | 55 | 1 | 2 | <1% | 3 | <0.1 |
| 00164 |  |  | Similar to SM30 | **S** | 93 | 1 | 24 | 5% | 4-7 | 3.6 |
| 07084 |  |  | Similar to Ctsd protein, partial; domain: partial Asp protease | **S** | 56 | 1 | 7 | 20% | 2-7 | 2.2 |
| 12385 |  |  | Similar to tetraspanin | **S** | 52 | 1 | 1 | 7% | 6 | 2.2 |
| 11223 | P07796 |  | Late histone H1-γ/H1b | **S** | 85 | 1 | 7 | 5% | 1-3,8,9 | 1.5 |
| 15123 |  |  | Similar to scavenger receptor Cys-rich, partial/Sp-SRCR-123 | **S** | 90 | 1 | 5 | 3% | 3,4,7 | 0.6 |
| 00475  25962 |  |  | Similar to MGC139263; domain: annexin | **S** | 90 | 1 | 2 | 9% | 5 | 0.5 |
| 10992  10240  10241  22528  08642 |  |  | Similar to scavenger receptor Cys-rich | **S** | 70 | 1 | 3 | 2% | 1,2,8 | 0.5 |
| 02359 |  |  | Similar to actin-bundling protein; domain: partial CH (calponin homology); N-term: N-acetylated Ala2 | **S** | 63 | 1 | 2 | 8% | 2,3 | 0.4 |
| 07157 |  |  | Hypothetical protein; domain: caveolin | **S** | 73 | 1 | 5 | 5% | 2,6,8,9 | 0.3 |
| 13893 |  |  | Similar to LOC494800 protein/Sp-CTs5; domain: Peptidase_C1A_cathepsinX | **S** | 60 | 1 | 1 | 3% | 6 | 0.3 |
| 12228 |  |  | Similar to MGC69420/actin-related protein 2/3 complex subunit 4 | **S** | 54 | 1 | 1 | 5% | 7 | 0.3 |
| 09046 |  |  | Similar to MGC86386 | **S** | 54 | 1 | 5 | 3% | 3-6 | 0.2 |
| 12097 |  |  | Similar to LOC495504/Sp-Sgsh (N-sulphoglucosamine sulfohydrolase; domain: sulfatase/arylsulfatase | **S** | 83 | 1 | 1 | 1% | 5 | 0.2 |
| 10169 |  |  | Hypothetical protein | **S** | 71 | 1 | 5 | 2% | 6,7 | 0.1 |
| 13016 |  |  | Similar to CG2082-PA; domain: Ndr | **S** | 124 | 1 | 2 | 5% | 2,6 | 0.1 |
| 13100 |  |  | Similar to arylsulfatase | **S** | 58 | 1 | 4 | 2% | 2-4 | 0.1 |
| 14208 |  |  | Hypothetical protein | **S** | 125 | 1 | 1 | 2% | 3 | 0.1 |
| 00281 |  |  | Similar to catalase | **S** | 69 | 1 | 1 | 3% | 2 | 0.1 |
| 05054  17449 |  |  | Similar to N-sulfoglucosamine sulfohydrolase (sulfamidase) | **S** | 74 | 1 | 1 | 2% | 3 | 0.1 |
| 13077 |  |  | Similar to TFP250/Similar to thrombospondin; domains: 3 CCP, 6 EGF_CA | **S** | 104 | 1 | 1 | <1% | 6 | <0.1 |
| 27046 |  |  | Similar to GA21473-PA, partial; domains: 3 IG | **S** | 48 | 1 | 1 | 1% | 5 | <0.1 |
| 15595 |  |  | Similar to calponin | **S** | 86 | 1 | 1 | 2% | 6 | <0.1 |
|  |  |  |  |  |  |  |  |  |  |  |

Identifications with a single unique peptide showing good quality, manually validated, spectra without MS^3^ confirmation. Tentatively identified proteins identified with high confidence in the respective other compartment were included in Additional file 1: Proteins identified in test and spines. Proteins are ordered according to decreasing emPAI in test. Proteins occurring in spines only are listed with decreasing emPAI in the second half of the table. The average absolute mass accuracy was 0.6ppm for test proteins and 1.4ppm for spine proteins (p<0.05). T, test; S, spines. Sequence coverage and emPAI were calculated for mature proteins. Unknown signal peptides were predicted (SignalP 3.0; [www.cbs.dtu.dk/services/SignalP](http://www.cbs.dtu.dk/services/SignalP)).
