# Supplementary material for: The sea urchin (Strongylocentrotus purpuratus) test and spine proteomes
Source: Proteome Sci. 2008 Aug 11;6:22. doi: 10.1186/1477-5956-6-22 (PMC2527298; doi:10.1186/1477-5956-6-22)
Supplement: Additional file 3 — Test and spine collagen peptides containing hydroxyproline. List of hydroxyproline-containing collagen peptides and frequency of occurrence. [file 1477-5956-6-22-S3.doc]

**Test and spine collagen peptides containing hydroxyproline**

|  |  |  |  |  |  |
| --- | --- | --- | --- | --- | --- |
| **GLEAN_3** | **Protein** |  | **Peptide** | **No. of HyP** | **Peptides** |
|  |  |  |  |  |  |
| **26008** | α1 collagen (1α) | **T** | _2586_GSDGS***P***GPVGA***P***GPAGPSGQ***P***GER_2609_ | 3 | 2 (2) |
|  |  | **T** | _2586_GSDGS***P***GPVGA***P***GPAG***P***SGQ***P***GER_2609_ | 4 | 2 (1) |
|  |  | **T** | _2673_GSEGSQGQTGP***P***GV***P***GR_2689_ | 2 | 16 |
|  |  | **T** | _2730_GDQGN***P***GQ***P***GAQGESGPLGPR_2750_ | 2 | 1 |
|  |  | **T**  **S** | _2751_GETGPAGP***P***GAQGESGER_2768_ | 1  1 | 12  1 |
|  |  | **T**  **S** | _2772_GSVGPAGP***P***GGVGER_2786_ | 1  1 | 8 (1)  2 |
|  |  | **T** | _2787_GPMGP***P***GMSGA***P***GA***P***GAK_2804_ | 3 | 6 |
|  |  | **T** | _2849_DGK***P***GPAGA***P***GE***P***GNSGPAGASGQR_2873_ | 3 | 3 (1) |
|  |  | **T** | _2874_GL***P***GLVGL***P***GPQGQR_2888_ | 2 | 13 (2) |
|  |  | **T** | _2892_GEDGGQGS***P***GA***P***GLTGE***P***GK_2911_ | 3 | 7 |
|  |  | **T** | _2892_GEDGGQGS***P***GA***P***GLTGE***P***GKR_2912_ | 3 | 6 |
|  |  | **T** | _2912_RGE***P***GVAGP***P***GPQGSAGER_2930_ | 2 | 10 (2) |
|  |  | **T**  **S** | _2913_GE***P***GVAGP***P***GPQGSAGER_2930_ | 2  2 | 9 (1)  1 |
|  |  | **T** | _3042_GP***P***GPSGS***P***GPDGPAGAEGDR_3062_ | 2 | 4 |
|  |  | **T** | _3096_GD***P***GDQGPQGS***P***GS***P***GFAGP***P***GR_3118_ | 4 | 12 |
|  |  | **T** | _3119_SGN***P***GPQGELGPTGAR_3134_ | 1 | 12 (2) |
|  |  | **T** | _3135_GETGG***P***GPSGPTGD***P***GPQGPLGA***P***GQQGER_3164_ | 3 | 4 |
|  |  | **T** | _3210_GE***P***GQSGS***P***GQ***P***GLAGTTGPSGER_3233_ | 3 | 11 |
|  |  | **T** | _3237_GNDGQSGP***P***GP***P***GPTGPAGQSGILGLAGGSGPR_3269_ | 2 | 1 |
|  |  | **T** | _3270_GPGGPAGP***P***GEAGSR_3284_ | 1 | 3 (1) |
|  |  | **T** | _3270_GPGG***P***AGP***P***GEAGSR_3284_  **(see Fig. 1)** | 2 | 1 (1) |
|  |  | **T**  **S** | _3363_GETGSTGA***P***GPQGPTGAR_3380_ | 1  1 | 10  2 |
|  |  | **T** | _3471_GEGGSSGP***P***GP***P***GP***P***GP***P***GP***P***GQVVQSSYGVR_3501_ | 5 | 2 |
| **26009** | α2 collagen (2α) | **T** | _2023_GEAGEGGNMGASGPVGAVGN***P***GQR_2046_ | 1 | 1 (1) |
| **05167** | α5 collagen (5α) | **T** | _1513_GAMAVGL***P***GSAGYAGTR_1529_ | 1 | 7 (1) |
|  |  | **T** | _1767_GN***P***GLIGFVGVDGPR_1781_ | 1 | 7 (1) |
|  |  | **T** | _1839_GTLGDNGEAGAAGT***P***GEAGTR_1859_ | 1 | 3 |
|  |  | **T** | _1908_GATGP***P***GQQGP***P***GLGGESGISGSR_1931_ | 2 | 4 |
|  |  | **T** | _1941_GEAGEEGE***P***GAAGQVGL***P***GSQGR_1963_ | 2 | 4 |
|  |  | **T** | _2042_QGEVGLTGSGGIGGP***P***GLR_2060_ | 1 | 5 (2) |
|  |  | **T** | _2061_GLQGP***P***GLR_2069_ | 1 | 1 (1) |
|  |  | **T** | _2070_GGVGPTGSMGEDGD***P***GVSGQQGSSGR_2095_ | 1 | 2 |
|  |  | **T** | _2100_GSQGP***P***GPTGPGGGR_2114_ | 1 | 5 (2) |
|  |  | **T** | _2148_GI***P***GN***P***GPGGA***P***GER_2162_ | 3 | 11 (1) |

T, test; S, spines. The prolines most likely hydroxylated according to the established collagen proline hydroxylation rules are in bold italics. Prolines confirmed to be hydroxylated by manual validation of selected spectra are underlined. The number of manually validated spectra of peptides is given in brackets after the total number of peptides with this sequence.
