# Supplementary material for: The sea urchin (Strongylocentrotus purpuratus) test and spine proteomes
Source: Proteome Sci. 2008 Aug 11;6:22. doi: 10.1186/1477-5956-6-22 (PMC2527298; doi:10.1186/1477-5956-6-22)
Supplement: Additional file 4 — Analysis of MSP130-related-3 and similar entries. Alignment of amino acid sequences of entries similar to MSP130-related-3. [file 1477-5956-6-22-S4.doc]

**Analysis of MSP130-related-3 and similar entries**

13823 1 MDTKLAYSAGKQWIHVVDFADMFFPKILDKFDSPRPVTDIAECGRYVAWA

06387 1 M--------GKQWIHVVDFADMFFPKILDKFDSPRPVTDIAECGRYVAWA

13823 51 VEGQEITDSGSVVLYDKFSRVDRKWNRNCEFIVGSRPKSIRFTKDCSTIV

06387 43 VEGQEITDSGSVVLYDKFSRVDRKWNRNCEFIVGSRPKSIRFTKDCSTIV

13823 101 VANEGVADVAGTGSASQWVNPEECIKGKAKDKEEEEMAETTGGTKEQTRI

06387 93 VANEGVADVAGTGSASQWVNPEE---------------------------

13823 151 IGVTQAGEDNGETMVKVDHPMEDQTTVNLIGITEEAMDGIIMDRIRDPIR

06387 --------------------------------------------------

13823 201 DPMGVARMGEEEAGITRVQTKGEMVPIKDQTQVDRDGTTKAQTRAQTKDQ

06387 --------------------------------------------------

13823 251 TKDRIEEELD------------------------GTTRDQIKEDGVPIKD

06387 116 ------EELDGTTKAQTKEVGYQTKGQTEVEVMAGTTRDQIKEDGVPIKD

13823 277 QTQVDRDGTIKAQTK-----------------------------------

06387 160 QTQEDRDGTIKAQTKAPTKALTKDQIEEELDGTTKAQTKEVGYQTKGRTE

13823 --------------------------------------------------

06387 210 VEVMAGTTKVQINEVEYPIRVQIKEVEYPLKVQIEEELGGTTKETTRDQT

13823 --------------------------------------------------

06387 260 KAQTKAPNQGPNRGGTGWNNQGPNQGGRVPNQGPNQGGRVPNQGPNQGGR

13823 --------------------------------------------------

06387 310 VPTQGPNRGGTGWNNQGNNQGPNQGPNQGPNQGPNRGGTGWNNQGPNQGG

13823 --------------------------------------------------

06387 360 RVPTQGPNRGGTGWNNQGNNQGPNQGPNQGPNRGGTGWNNQGPQGGGARG

13823 --------------------------------------------------

06387 410 NPQIPNHGGPNQGPNAGGTGWNRGQTNTPNARGRAPPNNPPNNPGQGPWQ

13823 292 -----------KRQYPPPPQTPNWQPQTPQWQPQTPQWQPQTPRWQPPQR

06387 460 IQHVLGHLIRQKRQYPPPPQTPNWQPQTPQWQPQTPQWQPQTPRWQPPQR

13823 331 PTFPQTPARGPGGGGHGMNPPNRGPGFNPGQGQGQTQWQRPGQRPGQRPG

06387 510 PTFPQTPARGPGGGGRGMNPPNSGPGFNPGQGQGQTQWQRPGQRPGQRPG

13823 381 QRPGQFPQVPGRGNTRDQNPQGQNPNGQPPQGGVTTSRGTCSGGGNFNPT

06387 560 QRPGQFPQVPGRGNTRDQNPQGQNPNGQSPQGGVTTSRGTCSGGGNFNPT

13823 431 VTTLDFTKFNARADWFKSQLVRQPYTGQMGDKQLNTFSQGLEPEYVTFDS

06387 610 VTTLDFTKFNARADWFKSQLVRQPYTGQMGDKQLNTFSQGLEPEYVTFDS

13823 481 METTAYVSLQENNAIASVDLFANEITGIHPLGAKQWKRYDLDPSPGNARG

06387 660 METTAYVSLQENNAIASVDLFANEITGIHPLGAKQWKRYDLDPSPGNARG

13823 531 FQKYDIESFRQPDAIESYTAANGETYIVTANEGKQLEYTCNLNACPPGGG

06387 710 FQKYDIESFRQPDAIESYTASNGETYIVTANEGKQLEYTCNLNACPPGGG

13823 581 EFVEFEKGDEFPEDYWLTAKLLERVSNAEMLDAFRLGNLEFSRIDGRSTE

06387 760 EFVEFEKGDEFPEDYWLTAKLLERVSNAEMLDAFRLGNLEFSRIDGRSTE

13823 631 QPLKHDDVYFYGGRGISAYRVDRQTGNLTLAWDSGDIIEKATAKYLPKMH

06387 810 QPLKHDDVYFYGGRGISAYRVDRQTGNLTLAWDSGDIIEKATAKYLPKMH

13823 681 NGNNRVGDPSLTMASTFDSQSDKMGPECESIEIGDVQGTKLIFVGIDRIS

06387 860 NGNNRVGDPSLTMASTFDSQSDKMGPECESIEIGDVQGTKLIFVGIDRIS

13823 731 AIALFSVPPDGNLPIFESIHRDGHIDKSFSELYRTKEFGDSDPESITFIP

06387 910 AIALFSVPPDGNLPIFESIHRDGHIDKSFSELYRTKEFGDSDPESIT---

13823 781 PEKSADKRPKLMVTGRVSGTITIYQIRDEPIWMLLKDGRSRDVRLLGSLH

06387 --------------------------------------------------

13823 831 VVLLSTFLAIVAGYRRLN

06387 ------------------

|  |
| --- |

Alignment of entries similar to MSP130 ([Glean3:13823]), MSP130 related-3 [19] and similar to MSP130” [Glean3:06387]. The Glean3:13823 sequence started at position 59 of the MSP130-3 sequence of [19] (see reference list of article). Peptides sequenced by MS/MS are shown in red.
