# Supplementary material for: The sea urchin (Strongylocentrotus purpuratus) test and spine proteomes
Source: Proteome Sci. 2008 Aug 11;6:22. doi: 10.1186/1477-5956-6-22 (PMC2527298; doi:10.1186/1477-5956-6-22)
Supplement: Additional file 5 — Sequences of unique peptides identified in spine matrix. List of sequences of accepted peptides from spine matrix. [file 1477-5956-6-22-S5.doc]

**Sequences of unique peptides identified in spine matrix**

| Description (ID) | Sequence |
| --- | --- |
| GLEAN3_00439 | NVDINPDVAAR |
| GLEAN3_00503 | VVLIGDSGVGK |
| GLEAN3_00513 | DLMALNIQR |
| GLEAN3_00513 | EDNIITEDLQR |
| GLEAN3_00513 | VETVQQEEAK |
| GLEAN3_00513 | VFGPLEFSR |
| GLEAN3_00513 | VGLLVSPDGK |
| GLEAN3_00552 | ILMVGLDAAGK |
| GLEAN3_00685 | DNIQGITKPAIR |
| GLEAN3_00685 | ISGLIYEETR |
| GLEAN3_00685 | TVTAMDVVYALK |
| GLEAN3_00685 | VFLENVIR |
| GLEAN3_01796 | ASNPVSAIAGEDYAGISR |
| GLEAN3_01796 | QADSDTFFSTTK |
| GLEAN3_01796 | SITLDSIYFGPGSR |
| GLEAN3_01796 | TGDLSIESSVR |
| GLEAN3_01796 | VANAIGAEPFTAR |
| GLEAN3_02503 | AGLQFPVGR |
| GLEAN3_02503 | HLQLAVR |
| GLEAN3_02503 | STELLIR |
| GLEAN3_02503 | YRPGTVALR |
| GLEAN3_03612 | DQSLYDQSLLK |
| GLEAN3_03918 | ANPEFGAPK |
| GLEAN3_03918 | IFDPSTSSEDYIK |
| GLEAN3_03918 | TQLQAENEVVPVVVTDK |
| GLEAN3_03918 | TTLGICSQR |
| GLEAN3_04721 | TITLEVEPSDSIENVK |
| GLEAN3_04721 | TLSDYNIQK |
| GLEAN3_04867 | IDPVVHDPNR |
| GLEAN3_04867 | MMVEPVPVWIGLHVGPMGR |
| GLEAN3_04867 | QPGFGNPGTPGGR |
| GLEAN3_04867 | QPGQPGVGGQPGVGGR |
| GLEAN3_04867 | QPGVGGQPGFGNPGTPGGR |
| GLEAN3_04867 | QPGVGGQPGVGGR |
| GLEAN3_04867 | QPGWGQPGVGQPGTPGGR |
| GLEAN3_04869 | AGLGAVLFDADLTVVPPNAIEISGK |
| GLEAN3_04876 | DALDLEK |
| GLEAN3_04876 | GGSIVFQDIK |
| GLEAN3_04876 | LMTFQNQR |
| GLEAN3_05538 | ITVGAFNGLSQLR |
| GLEAN3_05538 | LETLDLSR |
| GLEAN3_05538 | LTNLLQDAFR |
| GLEAN3_05872 | ILEVAPAPVSK |
| GLEAN3_05989 | ASPLAPR |
| GLEAN3_05989 | FQHNFLTFTGGANNK |
| GLEAN3_05989 | GYLITAK |
| GLEAN3_05989 | LPFMEAQMQCLSFR |
| GLEAN3_05989 | MPGQAVYPLQGCQPGWTNFGK |
| GLEAN3_05989 | VWMGLAER |
| GLEAN3_05990 | FKPDQPQQNAHR |
| GLEAN3_05990 | FSAQAVPGQRPGFGMPPR |
| GLEAN3_05990 | GHLIVTK |
| GLEAN3_05990 | IWMGLAELPSAPESNR |
| GLEAN3_05990 | LPYDEANMFCAR |
| GLEAN3_05991 | GYLMTSK |
| GLEAN3_05991 | MNWLQAQR |
| GLEAN3_05991 | TEMSFICQYQYML |
| GLEAN3_05991 | VWLGLSEK |
| GLEAN3_05991 | YSALNGTTPR |
| GLEAN3_05991 | YTGGGLNK |
| GLEAN3_06387 | DQNPQGQNPNGQSPQGGVTTSR |
| GLEAN3_06387 | GTCSGGGNFNPTVTTLDFTK |
| GLEAN3_06387 | ISAIALFSVPPDGNLPIFESIHR |
| GLEAN3_06387 | LGNLEFSR |
| GLEAN3_06387 | LIFVGIDR |
| GLEAN3_06387 | MGPECESIEIGDVQGTK |
| GLEAN3_06387 | QPYTGQMGDK |
| GLEAN3_06387 | VGDPSLTMASTFDSQSDK |
| GLEAN3_06387 | VPNQGPNQGGR |
| GLEAN3_06387 | VSNAEMLDAFR |
| GLEAN3_06387 | YDLDPSPGNAR |
| GLEAN3_07231 | DDIDKLVDDNMTDLQK |
| GLEAN3_07231 | LVDDNMTDLQK |
| GLEAN3_07682 | HLAQTYATK |
| GLEAN3_07682 | LLMPGLYTITATAPGYEPQSR |
| GLEAN3_07682 | SFYTASPDDAVFK |
| GLEAN3_08305 | VAQGSDVIK |
| GLEAN3_09481 | AGFAGDDAPR |
| GLEAN3_09481 | DLTDYLMK |
| GLEAN3_09481 | DLYANTVLSGGSTMFPGIADR |
| GLEAN3_09481 | DSYVGDEAQSK |
| GLEAN3_09481 | EITALAPPTMK |
| GLEAN3_09481 | GYSFTTTAER |
| GLEAN3_09481 | HQGVMVGMGQK |
| GLEAN3_09481 | IIAPPER |
| GLEAN3_09481 | QEYDESGPSIVHR |
| GLEAN3_09481 | SYELPDGQVITIGNER |
| GLEAN3_09549 | ASPGSITLAIR |
| GLEAN3_09549 | DSAGYVGFVFK |
| GLEAN3_09549 | DSPGAMGGLR |
| GLEAN3_09549 | SLYPSLEDMKVDK |
| GLEAN3_11106 | ALDALLS |
| GLEAN3_11106 | FGTDESEFQR |
| GLEAN3_11106 | FINDPIQYYVDK |
| GLEAN3_11106 | GLGTDEAVLVR |
| GLEAN3_11106 | LLVGLSVGGR |
| GLEAN3_11106 | QVFAAYGSLTSK |
| GLEAN3_11106 | TGYLNLVR |
| GLEAN3_11106 | TNAQIAEIK |
| GLEAN3_11106 | VQADAQALYEAGAAK |
| GLEAN3_11163 | GPNMCTVSFQNR |
| GLEAN3_11163 | QQVTDILNFNQMAR |
| GLEAN3_11180 | GVCTNDPFTGFK |
| GLEAN3_11180 | VQPGAGPGNNPNTGR |
| GLEAN3_11332 | ANLMSVVSR |
| GLEAN3_11332 | DVAILIPK |
| GLEAN3_11332 | FISESPEDSQVGIASYSNAGR |
| GLEAN3_11332 | GIWYVSIK |
| GLEAN3_11332 | GQGVEVQASEYGPPSSGR |
| GLEAN3_11332 | LLALDDVGNR |
| GLEAN3_11332 | LPGLIQK |
| GLEAN3_11332 | LQSPQTSSQTNPLLALR |
| GLEAN3_11332 | NPSGHVYTLR |
| GLEAN3_11332 | SPDFLGGANPPSADIVDTTPTFTLIR |
| GLEAN3_11332 | STTVAVVDTSR |
| GLEAN3_11332 | SYLQDMTPEGK |
| GLEAN3_11332 | VQWDQNPVTVELR |
| GLEAN3_11588 | LGSSFAFGK |
| GLEAN3_11588 | MGVFASDDAQGTSR |
| GLEAN3_11588 | NDEAPQVLDMGDR |
| GLEAN3_11588 | TGPGFTLK |
| GLEAN3_11588 | VGAFGSANSR |
| GLEAN3_12011 | EDVSQWVTSYK |
| GLEAN3_12011 | IFTGNVDR |
| GLEAN3_12011 | LACSTDGTTFHTVQGISTNPGADR |
| GLEAN3_12011 | NTIVTNTLPVPQICR |
| GLEAN3_12011 | VISVATQGR |
| GLEAN3_12112 | LVLLGESAVGK |
| GLEAN3_12518 | DMLPTDLSCFYR |
| GLEAN3_12518 | KQSPINIESR |
| GLEAN3_12518 | QSPINIESR |
| GLEAN3_12518 | TIAEAVK |
| GLEAN3_12518 | VEVSNDGHTLK |
| GLEAN3_12518 | VEYYAHLPLR |
| GLEAN3_12518 | VSTEGMYVLK |
| GLEAN3_12518 | YNGSLTVPK |
| GLEAN3_13301 | IGSVFESVNR |
| GLEAN3_13669 | GNDAVSNALQTESVFVDMQDIPDAAFIR |
| GLEAN3_13669 | IWEFSQGVYQPR |
| GLEAN3_13669 | RGNDAVSNALQTESVFVDMQDIPDAAFIR |
| GLEAN3_13669 | TSYFLIGGQVSTFK |
| GLEAN3_13670 | ADIIVQFAR |
| GLEAN3_13670 | DNEVFFIAETR |
| GLEAN3_13670 | FTLNTDDVR |
| GLEAN3_13670 | GGVSYFLIDK |
| GLEAN3_13670 | GIQSLYGAR |
| GLEAN3_13670 | GTEPVSNALR |
| GLEAN3_13670 | GYMQIYEYVEGAETPEELR |
| GLEAN3_13670 | KGTEPVSNALR |
| GLEAN3_13670 | LDFNPTR |
| GLEAN3_13670 | NGPINAAWTEGK |
| GLEAN3_13670 | SLMAPYYQGFQPR |
| GLEAN3_13670 | TEAVFTEMSAVPDAAFIR |
| GLEAN3_13670 | TNGIDAAFK |
| GLEAN3_13821 | DGHIDASYNALYK |
| GLEAN3_13821 | ELCSVEVGSQPIAVR |
| GLEAN3_13821 | FNAPAEVQR |
| GLEAN3_13821 | GPECESLAVGDVQGR |
| GLEAN3_13821 | GQTGGMYALNNGVAFK |
| GLEAN3_13821 | IDASSADR |
| GLEAN3_13821 | KYVNPEGTITTVR |
| GLEAN3_13821 | LPVGGGAGGAGGAGGAGGGGGGGGGAGGR |
| GLEAN3_13821 | LYLPFDKLPVGGGAGGAGGAGGAGGGGGGGGGAGGR |
| GLEAN3_13821 | QPYTGQLGDPGPHTFSR |
| GLEAN3_13821 | SQTNKPLLLVTGR |
| GLEAN3_13821 | TGDLSPESLSFIPPEK |
| GLEAN3_13821 | YVNPEGTITTVR |
| GLEAN3_13822 | CDTIIIANEGPAAENVEQEMFVNPEGTVSVVR |
| GLEAN3_13822 | ISLVDGLQEQGSEMIDRPVFFGGR |
| GLEAN3_13822 | LFNDPALAPLMEQQFIR |
| GLEAN3_13822 | TDPGMVHIYQK |
| GLEAN3_13822 | VIIVGIK |
| GLEAN3_13822 | VNPYGLDLVWDSGDSISK |
| GLEAN3_13822 | VSGTVNLYR |
| GLEAN3_13823 | EFGDSDPESITFIPPEK |
| GLEAN3_13823 | TKEFGDSDPESITFIPPEK |
| GLEAN3_13823 | VSGTITIYQIR |
| GLEAN3_13825 | IMLSYLTTVFGQGR |
| GLEAN3_13825 | MTFNEATFFCNR |
| GLEAN3_13825 | YGGSLYALDSPSK |
| GLEAN3_14496 | TFAQLLEDR |
| GLEAN3_14496 | VYDSGDEIER |
| GLEAN3_14564 | LLAQTTLR |
| GLEAN3_14564 | NLAEILAER |
| GLEAN3_14564 | VIAAEGEQNAAR |
| GLEAN3_14715 | AIQYIQSIK |
| GLEAN3_14715 | LPDTEVIR |
| GLEAN3_16506 | GVAETYPQVFNSK |
| GLEAN3_16506 | KAPMDGEFSILMDNK |
| GLEAN3_16506 | NMGDIDPAK |
| GLEAN3_16506 | NPQNPNSGYDYVSFFGGR |
| GLEAN3_16506 | SATPDNYNYLMVMGK |
| GLEAN3_16506 | SGTLSMYR |
| GLEAN3_16845 | AMSIMNSFVNDVFER |
| GLEAN3_16845 | LLLPGELAK |
| GLEAN3_16845 | QVHPDTGISSR |
| GLEAN3_18406 | FPNIGTGGYPGSVFPHGPGYPR |
| GLEAN3_18406 | GGGYGGAGGR |
| GLEAN3_18406 | GIHPAAGGPAYNGR |
| GLEAN3_18406 | GVGGAGGAGGGTGAAGR |
| GLEAN3_18406 | RPSASDSGSGGTGVNGGTGGGAR |
| GLEAN3_18406 | RTGVGILPDIQVIDPR |
| GLEAN3_18406 | STYPGQNYPGSR |
| GLEAN3_18406 | TGVGILPDIQVIDPR |
| GLEAN3_18406 | YPGYNPR |
| GLEAN3_18406 | YPNVGNPGMNYPGGYPGVGVGGFPGQGGYPGNNYPGQNYPGNNFPGSR |
| GLEAN3_18810 | ELGTIPGR |
| GLEAN3_18810 | LVASFSQDNQMER |
| GLEAN3_18810 | NPFGMPPGFAPVMR |
| GLEAN3_18810 | RELGTIPGR |
| GLEAN3_18810 | SPQENMEIYR |
| GLEAN3_18811 | AFVCEVPAGR |
| GLEAN3_18811 | NIPIGQQPGMGQGGFGNQQPGMGGR |
| GLEAN3_18811 | QIPQGVGPQWEAVEVTAMR |
| GLEAN3_18811 | QPGFGNQPGMGGR |
| GLEAN3_18811 | QPGFGNQPGVGGR |
| GLEAN3_18811 | QPGMGGQPGVGGR |
| GLEAN3_18811 | QPGMGGQQPGMGGQPGVGGR |
| GLEAN3_18811 | QPGMGGQQPGWGNQPGVGGR |
| GLEAN3_18811 | QPGMGGQQPNNPNNPNPNNPNNPNNPNPR |
| GLEAN3_18811 | QPGWGNQPGVGGR |
| GLEAN3_18811 | SWPVNPQNPMSGPPGR |
| GLEAN3_18813 | CMTLPGQMQMNGAVQK |
| GLEAN3_18813 | GGWGQGGQGQGGQGGR |
| GLEAN3_18813 | TFTGCDGISPGHLAAPTTFEER |
| GLEAN3_18813 | TYDMASQSCK |
| GLEAN3_18813 | WNPNQGAGAGAGAGGR |
| GLEAN3_18813 | WNPQTPQNPGQGGR |
| GLEAN3_19691 | IAAEIAAPLSK |
| GLEAN3_19691 | IDTDNVYTR |
| GLEAN3_19967 | IISIATQGR |
| GLEAN3_19967 | LSGIVTQGR |
| GLEAN3_19967 | VAGIITQGR |
| GLEAN3_21260 | VISSLSSNYK |
| GLEAN3_21260 | VLAATLGAYR |
| GLEAN3_21385 | FATTQGNCAAQFGHR |
| GLEAN3_21385 | FIQVVDYSR |
| GLEAN3_21385 | GTPAADAATNTFTDPEGTISIVR |
| GLEAN3_21385 | MTVTTIDFR |
| GLEAN3_21385 | SFTDAVPFSR |
| GLEAN3_21385 | TTAPAADIAR |
| GLEAN3_21385 | VGADPVSLK |
| GLEAN3_21385 | YDQGAANSVAFDPASSFAYVAGNK |
| GLEAN3_22047 | VDCAESLCR |
| GLEAN3_22366 | ILSPNYPR |
| GLEAN3_22366 | LVIDSPGGSTYR |
| GLEAN3_22366 | TAEGSTLSASFK |
| GLEAN3_22561 | FITDVDDNDAALMER |
| GLEAN3_22561 | LLANNYDVSVNR |
| GLEAN3_23016 | FDITDGTNPLIDR |
| GLEAN3_23016 | GHLESTDNPGMPINSFTQLDLAGSK |
| GLEAN3_23016 | GVEPEEDQFTFR |
| GLEAN3_23016 | IIDQPLLR |
| GLEAN3_23016 | IYYVHTAEDEIR |
| GLEAN3_23016 | LITVDEGGQR |
| GLEAN3_23016 | NQPITSFTQK |
| GLEAN3_23016 | QSEPLSQIPGR |
| GLEAN3_23016 | VSYRPPSTELGIAPR |
| GLEAN3_23016 | VTVLSSSSGLPR |
| GLEAN3_23052 | DFSQLPNIDEVR |
| GLEAN3_23052 | NQLLAQTASK |
| GLEAN3_23115 | AAAPGQVMVVDVMER |
| GLEAN3_23115 | TLDSIELR |
| GLEAN3_24352 | ASFVDMR |
| GLEAN3_24352 | NSFQAVMVTDGR |
| GLEAN3_24352 | VAFFGAADTSIR |
| GLEAN3_24352 | VDQFTPDPFPLADGR |
| GLEAN3_24565 | LNELQTVEDEGR |
| GLEAN3_24565 | TATVNFAIEPTK |
| GLEAN3_24730 | QNYHEESEAGVNR |
| GLEAN3_25966 | CAFPAITTLPK |
| GLEAN3_25966 | ELFLQDNLINSVSR |
| GLEAN3_25966 | GAFSSMTQLQTLR |
| GLEAN3_25966 | LDINFLK |
| GLEAN3_25966 | TLQLADNPLER |
| GLEAN3_25966 | TVSTGATFILPCTLTAQAAAR |
| GLEAN3_25966 | VDGAMFSR |
| GLEAN3_25966 | VFYSLVGDR |
| GLEAN3_26000 | NQPDTVTVYAGDTIYMR |
| GLEAN3_26000 | SDNTNPQTQYISIR |
| GLEAN3_26000 | SEFTVIR |
| GLEAN3_26000 | YSDGSIFFTR |
| GLEAN3_26008 | GEPGVAGPPGPQGSAGER |
| GLEAN3_26008 | GETGPAGPPGAQGESGER |
| GLEAN3_26008 | GETGSTGAPGPQGPTGAR |
| GLEAN3_26008 | GSTGPAGPSGPSGPAGER |
| GLEAN3_26008 | GSVGPAGPPGGVGER |
| GLEAN3_26843 | FDILGTVASK |
| GLEAN3_26843 | QLHEVSGIMSQGR |
| GLEAN3_26843 | TNTPDQFIQADLR |
| GLEAN3_26949 | ATDSTLTLETLK |
| GLEAN3_26949 | DSTQMLVDVGTQDTTEK |
| GLEAN3_26949 | EYGLVPIVQETYAQDR |
| GLEAN3_26949 | GANLTPQISCYEETSK |
| GLEAN3_26949 | GLMSSDFR |
| GLEAN3_26949 | LLLDGQEQFGSDDGAVFK |
| GLEAN3_26949 | MFDSQAYSDTDLLFK |
| GLEAN3_26949 | MPITAWETCNLAK |
| GLEAN3_26949 | NANEPYYDYAGAFR |
| GLEAN3_26949 | QCPADSSELYQSYAGAFR |
| GLEAN3_26949 | TAFSAQSLSPEVVCVAGSGISDCLMK |
| GLEAN3_26949 | TYLGDYANTIDGLK |
| GLEAN3_26949 | VPSHAVVTSSTK |
| GLEAN3_26949 | VQDGQAHMITLDGGDVYLAGK |
| GLEAN3_26949 | YAGIAVVR |
| GLEAN3_27145 | FSDPDTLDEEVVNVR |
| GLEAN3_27169 | DNIVIGGQAGVYDPNR |
| GLEAN3_27169 | GNFGGNLFWIK |
| GLEAN3_27169 | HMPDKFNVAR |
| GLEAN3_27169 | HQNVAEIINVNSPVELVTAPLIR |
| GLEAN3_27169 | IPDYWVVDNER |
| GLEAN3_27169 | LIMVVNMGTSGQVPNR |
| GLEAN3_27169 | MQAPAFGLASTTFK |
| GLEAN3_27169 | MVIINANIQNAGVYR |
| GLEAN3_27236 | AVPSSYSDLGK |
| GLEAN3_27236 | ITLSTLIDGK |
| GLEAN3_27236 | LTLDTSFSPQTGK |
| GLEAN3_27236 | YVLDSEASLNAK |
| GLEAN3_27372 | GYLNDASDFVAR |
| GLEAN3_27372 | LVGGSNEAEGR |
| GLEAN3_27906 | LLDIADFDSFR |
| GLEAN3_27906 | NLYTHTLPFYQPPTGQQLDFIPPEK |
| GLEAN3_27906 | NPGNPNQPVR |
| GLEAN3_28135 | ADTDAAPAAPAPSTPK |
| GLEAN3_28135 | GVTSGQLR |
| GLEAN3_28748 | FDELEQR |
| GLEAN3_28748 | TYDAITLIR |
| GLEAN3_28749 | GAPSNIDAIFEKPGGTTVMIK |
| GLEAN3_28749 | MGLSNGISAAFSWPQDR |
| GLEAN3_28749 | NSQLVQGYPVR |
| GLEAN3_28749 | NVLEDIPGLPLGIDAAFSSK |
| GLEAN3_28749 | TASYFVR |
| GLEAN3_28749 | TTITFTFDNYTPDLPMNQVR |
| GLEAN3_28749 | YDHSSGSLSQGFPR |
| GLEAN3_28749 | YVLSGASWGR |

The peptides are arranged according to increasing Glean3 entry number of the corresponding proteins. Peptides shared with human proteins are shaded yellow.
