# Supplementary material for: The sea urchin (Strongylocentrotus purpuratus) test and spine proteomes
Source: Proteome Sci. 2008 Aug 11;6:22. doi: 10.1186/1477-5956-6-22 (PMC2527298; doi:10.1186/1477-5956-6-22)
Supplement: Additional file 6 — Sequences of unique peptides identified in test matrix. List of sequences of accepted peptides from test matrix. [file 1477-5956-6-22-S6.doc]

**Sequences of unique peptides identified in test matrix**

| Description (ID) | Sequence |
| --- | --- |
| GLEAN3_00061 | AILVDLEPGTMDSVR |
| GLEAN3_00062 | ALTVPELTQQMFDAK |
| GLEAN3_00062 | AVLVDLEPGTMDSVR |
| GLEAN3_00062 | EVDEQMLNVQNK |
| GLEAN3_00062 | GHYTEGAELVDSVLDVVRK |
| GLEAN3_00062 | IMNTFSVVPSPK |
| GLEAN3_00062 | INVYYNEATGGK |
| GLEAN3_00062 | LAVNMVPFPR |
| GLEAN3_00062 | LHFFMPGFAPLTSR |
| GLEAN3_00062 | LTTPTYGDLNHLVSATMSGVTTCLR |
| GLEAN3_00062 | NSSYFVEWIPNNVK |
| GLEAN3_00062 | SGPFGQIFRPDNFVFGQSGAGNNWAK |
| GLEAN3_00379 | KLYDIDVAK |
| GLEAN3_00379 | LAPDYDALDVANK |
| GLEAN3_00438 | TNDAFVAIPTDALGK |
| GLEAN3_00439 | LSLLIGANEPGLTR |
| GLEAN3_00439 | NVDINPDVAAR |
| GLEAN3_00439 | TFTADATVPLDDVR |
| GLEAN3_00469 | ATGSPPPTVEWLYR |
| GLEAN3_00469 | FVQVPPSVLDR |
| GLEAN3_00469 | SDSLIINQISK |
| GLEAN3_00469 | TDAGFYTASVTDNTR |
| GLEAN3_00469 | TPIQSQCTPDQLR |
| GLEAN3_00469 | VSQQDLGTYICK |
| GLEAN3_00469 | VTEQGTADPEVIQAETQVLFAVPVALYPR |
| GLEAN3_00595 | IGGIGTVPVGR |
| GLEAN3_00595 | NMITGTSQADCAVLVVAAGIGEFEAGISK |
| GLEAN3_00595 | STTTGHLIYK |
| GLEAN3_00685 | ISGLIYEETR |
| GLEAN3_00685 | TVTAMDVVYALK |
| GLEAN3_00685 | VFLENVIR |
| GLEAN3_01796 | TGDLSIESSVR |
| GLEAN3_01892 | LLDVDSDR |
| GLEAN3_01892 | TTNEALAVFEGIR |
| GLEAN3_03825 | DSTLIMQLLR |
| GLEAN3_03825 | IVSSIEQK |
| GLEAN3_03825 | LAEQAER |
| GLEAN3_03825 | NLLSVAYK |
| GLEAN3_03918 | TPDGIAVDWINK |
| GLEAN3_04721 | ESTLHLVLR |
| GLEAN3_04721 | TITLEVEPSDSIENVK |
| GLEAN3_04721 | TLSDYNIQK |
| GLEAN3_04746 | IGAPAIAYMTPTIATTIGSTVR |
| GLEAN3_04746 | NSLAVISKPMSLNVK |
| GLEAN3_04746 | SNPGQLLPSGR |
| GLEAN3_04867 | APMAEPGLGAMIFDADIINQIFNNQVEITPQWVPEQGR |
| GLEAN3_04867 | FWMEEGNSCYLFDSGAFLR |
| GLEAN3_04867 | IDPVVHDPNR |
| GLEAN3_04867 | MMVEPVPVWIGLHVGPMGR |
| GLEAN3_04867 | NEPHALICEYHPQDLLPGNYPHGNYPPR |
| GLEAN3_04867 | QPGFGNPGTPGGR |
| GLEAN3_04867 | QPGQPGVGGQPGVGGR |
| GLEAN3_04867 | QPGVGGQPGFGNPGTPGGR |
| GLEAN3_04867 | QPGVGGQPGVGGR |
| GLEAN3_04867 | QPGWGQPGVGQPGTPGGR |
| GLEAN3_05014 | VEIFLGNK |
| GLEAN3_05032 | DGDGTITTK |
| GLEAN3_05032 | EAFSLFDK |
| GLEAN3_05167 | GAAGLQGPGGLVGER |
| GLEAN3_05167 | GAMAVGLPGSAGYAGTR |
| GLEAN3_05167 | GATGPPGQQGPPGLGGESGISGSR |
| GLEAN3_05167 | GDRGPIGQTGTMGATGEVGEQGLR |
| GLEAN3_05167 | GEAGEEGEPGAAGQVGLPGSQGR |
| GLEAN3_05167 | GGVGPTGSMGEDGDPGVSGQQGSSGR |
| GLEAN3_05167 | GIPGNPGPGGAPGER |
| GLEAN3_05167 | GLQGPPGLR |
| GLEAN3_05167 | GNPGLIGFVGVDGPR |
| GLEAN3_05167 | GPIGQTGTMGATGEVGEQGLR |
| GLEAN3_05167 | GPSGQSGPSGPIGSAGPR |
| GLEAN3_05167 | GSEGIAGIAGPR |
| GLEAN3_05167 | GSQGPPGPTGPGGGR |
| GLEAN3_05167 | GTLGDNGEAGAAGTPGEAGTR |
| GLEAN3_05167 | GVLGALGAQGER |
| GLEAN3_05167 | QGEVGLTGSGGIGGPPGLR |
| GLEAN3_05167 | VGSQGESGEQGLTGPTGSR |
| GLEAN3_05238 | MGIADAFYIDQK |
| GLEAN3_05238 | QIPIPIASISDEAHPMK |
| GLEAN3_05385 | ELGLPADIDGALPWGQTGK |
| GLEAN3_05385 | GQVDLGFPR |
| GLEAN3_05385 | ILNFTPDLTEAEVVDSFER |
| GLEAN3_05385 | SFNAVAFIR |
| GLEAN3_05385 | VWSDVTPLTFR |
| GLEAN3_05538 | IESISASTFSGAAK |
| GLEAN3_05538 | LTNLLQDAFR |
| GLEAN3_05989 | ASPLAPR |
| GLEAN3_05989 | FQHNFLTFTGGANNK |
| GLEAN3_05989 | GYLITAK |
| GLEAN3_05989 | LPFMEAQMQCLSFR |
| GLEAN3_05989 | MPGQAVYPLQGCQPGWTNFGK |
| GLEAN3_05989 | VWMGLAER |
| GLEAN3_05990 | FKPDQPQQNAHR |
| GLEAN3_05990 | FSAQAVPGQRPGFGMPPR |
| GLEAN3_05990 | GHLIVTK |
| GLEAN3_05990 | IWMGLAELPSAPESNR |
| GLEAN3_05990 | LPYDEANMFCAR |
| GLEAN3_05990 | YFWADGTEFFFTR |
| GLEAN3_05991 | MNWLQAQR |
| GLEAN3_05992 | ELQTWVTSNWR |
| GLEAN3_05992 | TPMSYVCK |
| GLEAN3_05992 | VDQPQLNWHR |
| GLEAN3_06211 | LVQAFQYTDK |
| GLEAN3_06387 | DQNPQGQNPNGQSPQGGVTTSR |
| GLEAN3_06387 | GGTGWNNQGNNQGPNQGPNQGPNQGPNR |
| GLEAN3_06387 | GGTGWNNQGNNQGPNQGPNQGPNR |
| GLEAN3_06387 | GGTGWNNQGPNQGGR |
| GLEAN3_06387 | GGTGWNNQGPQGGGAR |
| GLEAN3_06387 | VPNQGPNQGGR |
| GLEAN3_06812 | CVYGNQFISAR |
| GLEAN3_06812 | NPPMVTVTPPEVNIK |
| GLEAN3_07682 | LLMPGLYTITATAPGYEPQSR |
| GLEAN3_07682 | SFYTASPDDAVFK |
| GLEAN3_07682 | VYTIGTSVK |
| GLEAN3_08560 | FEELNMDLFR |
| GLEAN3_08560 | GVPQIEVTFEIDVNGILR |
| GLEAN3_08560 | ITITNDQNR |
| GLEAN3_08560 | NQLTSNPENTVFDAK |
| GLEAN3_09165 | LLQDFFNGK |
| GLEAN3_09165 | SINPDEAVAYGAAVQAAILSGDK |
| GLEAN3_09477 | DAGVIAGLNVLR |
| GLEAN3_09477 | IINEPTAAALAYGLDK |
| GLEAN3_09477 | STAGDTHLGGEDFDDR |
| GLEAN3_09477 | TTPSYVAFTDTER |
| GLEAN3_09477 | VEIIANDQGNR |
| GLEAN3_09549 | ASPGSITLAIR |
| GLEAN3_10169 | ATFTCALMSTNNIGVR |
| GLEAN3_10169 | GEEELEENEGLR |
| GLEAN3_10169 | GYSWNVEPSVTDMHQSSPGR |
| GLEAN3_10169 | QILRPQDTNIQYTCLMR |
| GLEAN3_10169 | VLEIQDIR |
| GLEAN3_11163 | GPNMCTVSFQNR |
| GLEAN3_11163 | QQVTDILNFNQMAR |
| GLEAN3_11180 | GKGPIVVEPVGPR |
| GLEAN3_11180 | GPIVVEPVGPR |
| GLEAN3_11180 | GVCTNDPFTGFK |
| GLEAN3_11180 | VQPGAGPGNNPNTGR |
| GLEAN3_11332 | ANLMSVVSR |
| GLEAN3_11332 | DVAILIPK |
| GLEAN3_11332 | FISESPEDSQVGIASYSNAGR |
| GLEAN3_11332 | GQGVEVQASEYGPPSSGR |
| GLEAN3_11332 | ISDLEVLGFDTDAGTAR |
| GLEAN3_11332 | LLALDDVGNR |
| GLEAN3_11332 | LQSPQTSSQTNPLLALR |
| GLEAN3_11332 | NMEMEYQSVELDVYSAAADPTQLPIR |
| GLEAN3_11332 | NPSGHVYTLR |
| GLEAN3_11332 | SPDFLGGANPPSADIVDTTPTFTLIR |
| GLEAN3_11332 | STTVAVVDTSR |
| GLEAN3_11332 | SYLQDMTPEGK |
| GLEAN3_11332 | TVIVIDQSATMGDDEVWQDVVR |
| GLEAN3_11332 | VQWDQNPVTVELR |
| GLEAN3_12518 | DMLPTDLSCFYR |
| GLEAN3_12518 | KQSPINIESR |
| GLEAN3_12518 | QSPINIESR |
| GLEAN3_12518 | VEVSNDGHTLK |
| GLEAN3_12518 | VEYYAHLPLR |
| GLEAN3_12518 | VEYYAHLPLRDMLPTDLSCFYR |
| GLEAN3_12518 | VSTEGMYVLK |
| GLEAN3_12549 | FADLQMDR |
| GLEAN3_12549 | GLPTNLDAAFYYEK |
| GLEAN3_12549 | LDAAFQFENK |
| GLEAN3_12549 | YNFLSSSVDPGYPK |
| GLEAN3_13301 | GIAENIPEVK |
| GLEAN3_13301 | GVQDTIGPEGK |
| GLEAN3_13301 | IGSVFESVNR |
| GLEAN3_13301 | LDSDLLLGR |
| GLEAN3_13301 | VGFTYNDLR |
| GLEAN3_13301 | YVLTGEQDIYR |
| GLEAN3_13669 | DNEVYFVTPK |
| GLEAN3_13669 | IWEFSQGVYQPR |
| GLEAN3_13670 | ADIIVQFAR |
| GLEAN3_13670 | DNEVFFIAETR |
| GLEAN3_13670 | FTLNTDDVR |
| GLEAN3_13670 | GGVSYFLIDK |
| GLEAN3_13670 | GIQSLYGAR |
| GLEAN3_13670 | GTEPVSNALR |
| GLEAN3_13670 | GYMQIYEYVEGAETPEELR |
| GLEAN3_13670 | KGTEPVSNALR |
| GLEAN3_13670 | NGPINAAWTEGK |
| GLEAN3_13670 | QVSTFKDNEVFFIAETR |
| GLEAN3_13670 | SLMAPYYQGFQPR |
| GLEAN3_13670 | TEAVFTEMSAVPDAAFIR |
| GLEAN3_13670 | TNGIDAAFK |
| GLEAN3_13670 | VLTTAFQVWGDVAR |
| GLEAN3_13670 | YAIQHGWSK |
| GLEAN3_13819 | SGETEDTFIADLVVGLCTGQIK |
| GLEAN3_13819 | VNQIGSVTESIEACK |
| GLEAN3_13821 | DGHIDASYNALYK |
| GLEAN3_13821 | ELCSVEVGSQPIAVR |
| GLEAN3_13821 | FNAPAEVQR |
| GLEAN3_13821 | GPECESLAVGDVQGR |
| GLEAN3_13821 | GQTGGMYALNNGVAFK |
| GLEAN3_13821 | IDASSADR |
| GLEAN3_13821 | IFNGAAFSRPPQR |
| GLEAN3_13821 | KTGDLSPESLSFIPPEK |
| GLEAN3_13821 | KYVNPEGTITTVR |
| GLEAN3_13821 | LPVGGGAGGAGGAGGAGGGGGGGGGAGGR |
| GLEAN3_13821 | LYLPFDKLPVGGGAGGAGGAGGAGGGGGGGGGAGGR |
| GLEAN3_13821 | QIATEGPVADIAECGDLVAFTQPGKPHFTDVGSLK |
| GLEAN3_13821 | QLAYVGGGQFVQIVDFSDVVQPK |
| GLEAN3_13821 | QPYTGQLGDPGPHTFSR |
| GLEAN3_13821 | SQTNKPLLLVTGR |
| GLEAN3_13821 | TGDLSPESLSFIPPEK |
| GLEAN3_13821 | YVNPEGTITTVR |
| GLEAN3_13822 | ADVLKPVGDHGIGNLDPDSIQFVPADR |
| GLEAN3_13822 | CDTIIIANEGPAAENVEQEMFVNPEGTVSVVR |
| GLEAN3_13822 | ISLVDGLQEQGSEMIDRPVFFGGR |
| GLEAN3_13822 | LFNDPALAPLMEQQFIR |
| GLEAN3_13822 | NWTNYGIDASTMDAK |
| GLEAN3_13822 | SRPPMVLVTSR |
| GLEAN3_13822 | TDPGMVHIYQK |
| GLEAN3_13822 | VIIVGIK |
| GLEAN3_13822 | VLDPIIDIAECGNLVAFTSRPR |
| GLEAN3_13822 | VNPYGLDLVWDSGDSISK |
| GLEAN3_13822 | VSGTVNLYR |
| GLEAN3_13823 | EFGDSDPESITFIPPEK |
| GLEAN3_13823 | GDEFPEDYWLTAK |
| GLEAN3_13823 | GTCSGGGNFNPTVTTLDFTK |
| GLEAN3_13823 | ILDKFDSPRPVTDIAECGR |
| GLEAN3_13823 | ISAIALFSVPPDGNLPIFESIHR |
| GLEAN3_13823 | LGNLEFSR |
| GLEAN3_13823 | LIFVGIDR |
| GLEAN3_13823 | MGPECESIEIGDVQGTK |
| GLEAN3_13823 | NCEFIVGSRPK |
| GLEAN3_13823 | QLEYTCNLNACPPGGGEFVEFEK |
| GLEAN3_13823 | STEQPLKHDDVYFYGGR |
| GLEAN3_13823 | TKEFGDSDPESITFIPPEK |
| GLEAN3_13823 | VGDPSLTMASTFDSQSDK |
| GLEAN3_13823 | VSGTITIYQIR |
| GLEAN3_13823 | VSNAEMLDAFR |
| GLEAN3_13823 | YDLDPSPGNAR |
| GLEAN3_13825 | AENALGWR |
| GLEAN3_13825 | IMLSYLTTVFGQGR |
| GLEAN3_13825 | MTFNEATFFCNR |
| GLEAN3_13825 | QKFWTGFYK |
| GLEAN3_13825 | YGGSLYALDSPSK |
| GLEAN3_13825 | YGGSLYALDSPSKNR |
| GLEAN3_14496 | IDGDFANPSFGFK |
| GLEAN3_14496 | LEFSVTDGLSK |
| GLEAN3_14869 | GLFIIDDK |
| GLEAN3_14869 | LVQAFQFTDK |
| GLEAN3_16506 | ANNAVVQLILTPGNEAITGFNPMGMK |
| GLEAN3_16506 | APMDGEFSILMDNK |
| GLEAN3_16506 | AQQQQQQGGQPNYPGQGAGAGTR |
| GLEAN3_16506 | FATTQGNCAAQFGHR |
| GLEAN3_16506 | FFHNCETIVIANK |
| GLEAN3_16506 | GTPAADAATNTFTDPEGTISIVR |
| GLEAN3_16506 | GVAETYPQVFNSK |
| GLEAN3_16506 | KAPMDGEFSILMDNK |
| GLEAN3_16506 | KSGTLSMYR |
| GLEAN3_16506 | KWNLLCDAK |
| GLEAN3_16506 | MTVTTIDFR |
| GLEAN3_16506 | NMGDIDPAK |
| GLEAN3_16506 | NPQNPNSGYDYVSFFGGR |
| GLEAN3_16506 | NQYNLASYLFTADEGATTSYR |
| GLEAN3_16506 | QPNAIESFEIR |
| GLEAN3_16506 | SATPDNYNYLMVMGK |
| GLEAN3_16506 | SFTDAVPFSR |
| GLEAN3_16506 | SGTLSMYR |
| GLEAN3_16506 | STTADAQSHGPK |
| GLEAN3_16506 | TWFNNDLDASSTPR |
| GLEAN3_16506 | VDGLRNPQNPNSGYDYVSFFGGR |
| GLEAN3_16506 | VGADPVSLK |
| GLEAN3_18406 | FPNIGTGGYPGSVFPHGPGYPR |
| GLEAN3_18406 | GVGGAGGAGGGTGAAGR |
| GLEAN3_18406 | RPSASDSGSGGTGVNGGTGGGAR |
| GLEAN3_18406 | RTGVGILPDIQVIDPR |
| GLEAN3_18406 | STYPGQNYPGSR |
| GLEAN3_18406 | TGVGILPDIQVIDPR |
| GLEAN3_18406 | YPNVGNPGMNYPGGYPGVGVGGFPGQGGYPGNNYPGQNYPGNNFPGSR |
| GLEAN3_18810 | LVASFSQDNQMER |
| GLEAN3_18810 | NPFGMPPGFAPVMR |
| GLEAN3_18810 | SPQENMEIYR |
| GLEAN3_18811 | AFVCEVPAGR |
| GLEAN3_18811 | MASEFCEMVTPCGNGPAK |
| GLEAN3_18811 | NIPIGQQPGMGQGGFGNQQPGMGGR |
| GLEAN3_18811 | QIPQGVGPQWEAVEVTAMR |
| GLEAN3_18811 | QPGFGNQPGMGGQQPGMGGQQPGWGNQPGVGGR |
| GLEAN3_18811 | QPGFGNQPGMGGR |
| GLEAN3_18811 | QPGFGNQPGVGGR |
| GLEAN3_18811 | QPGMGGQPGVGGR |
| GLEAN3_18811 | QPGMGGQQPGMGGQPGVGGR |
| GLEAN3_18811 | QPGMGGQQPGWGNQPGVGGR |
| GLEAN3_18811 | QPGMGGQQPNNPNNPNPNNPNNPNNPNPR |
| GLEAN3_18811 | QPGVGGR |
| GLEAN3_18811 | QPGWGNQPGVGGR |
| GLEAN3_18811 | SWPVNPQNPMSGPPGR |
| GLEAN3_18813 | AFGQLKTYDMASQSCK |
| GLEAN3_18813 | GGWGAGAGTGQGAGGGWGGQNPQNPGAGGGR |
| GLEAN3_18813 | GGWGQGGQGQGGQGGR |
| GLEAN3_18813 | IRFDLLRPIGGNR |
| GLEAN3_18813 | LRPSNVWMGFR |
| GLEAN3_18813 | TFTGCDGISPGHLAAPTTFEER |
| GLEAN3_18813 | TFTGCDGISPGHLAAPTTFEERR |
| GLEAN3_18813 | TYDMASQSCK |
| GLEAN3_18813 | VENSCYR |
| GLEAN3_18813 | WNPNQGAGAGAGAGGR |
| GLEAN3_18813 | WNPNQGAGAGAGAGGRWNPNQGAGAGAGAGGR |
| GLEAN3_18813 | WNPQTPQNPGQGGR |
| GLEAN3_20322 | ELISNSSDALDK |
| GLEAN3_20322 | EMLQQSK |
| GLEAN3_20322 | GVVDSEDLPLNISR |
| GLEAN3_20322 | SLTNDWEDHLAVK |
| GLEAN3_20457 | FTCVATNPALQQPSTCSLTPLR |
| GLEAN3_20457 | GGDPPATLSWVR |
| GLEAN3_20457 | LNPYGQVGEEQLSR |
| GLEAN3_21260 | VISSLSSNYK |
| GLEAN3_21260 | VLAATLGAYR |
| GLEAN3_22047 | DAAVCFSQNPFQNR |
| GLEAN3_22047 | HAFITTYAVEMK |
| GLEAN3_22047 | VDCAESLCR |
| GLEAN3_22366 | CGEDIDLSER |
| GLEAN3_22366 | EVLTYANNLWVR |
| GLEAN3_22366 | GDTMWISFR |
| GLEAN3_22366 | GWLINFADAK |
| GLEAN3_22366 | HSGPELPDPTSFK |
| GLEAN3_22366 | ILSPNYPR |
| GLEAN3_22366 | ILVNFLEFNTER |
| GLEAN3_22366 | LFFPQFQTEQYR |
| GLEAN3_22366 | LVIDSPGGSTYR |
| GLEAN3_22366 | RCGEDIDLSER |
| GLEAN3_22366 | SASGSGLPEPAGFDTPSNEAWIYFR |
| GLEAN3_22366 | SPFPDAR |
| GLEAN3_22366 | TAEGSTLSASFK |
| GLEAN3_22366 | VNLNCIWK |
| GLEAN3_22366 | YDTVSAGYGSSPAR |
| GLEAN3_22631 | LIYDTEFK |
| GLEAN3_23052 | CYQCFGLGSR |
| GLEAN3_23052 | DFSQLPNIDEVR |
| GLEAN3_23115 | AAAPGQVMVVDVMER |
| GLEAN3_23115 | GYGPFSQEISR |
| GLEAN3_23115 | HGDIVHYEYQLLDAK |
| GLEAN3_23115 | LSNLEPYSTYDVYVQAVSDAGMAPVATR |
| GLEAN3_23115 | TLDSIELR |
| GLEAN3_23115 | WNTLECGDR |
| GLEAN3_23217 | FNVWDTAGQEK |
| GLEAN3_23217 | LVLVGDGGTGK |
| GLEAN3_24082 | EQVANSAFVER |
| GLEAN3_24103 | KPLVIIAEDVDGEALSTLVLNR |
| GLEAN3_24103 | LSDGVAVLK |
| GLEAN3_24103 | VTDALNATR |
| GLEAN3_24564 | IDEYVLPK |
| GLEAN3_24564 | IVFQDSISR |
| GLEAN3_24565 | LLLVEPEGVAQEYVVSDLVDFSNAAAYSK |
| GLEAN3_24565 | LNELQTVEDEGR |
| GLEAN3_24565 | SFLQAAPYIYIDPAK |
| GLEAN3_24565 | STANTALTNAMNYLVSR |
| GLEAN3_24565 | TATVNFAIEPTK |
| GLEAN3_24565 | TSDILSDTTK |
| GLEAN3_24565 | YLTETGQGNPTIEQK |
| GLEAN3_24890 | AGLQFPVGR |
| GLEAN3_24890 | HLQLAIR |
| GLEAN3_24890 | VGATAAVYSAAILEYLTAEVLELAGNASK |
| GLEAN3_25068 | YNTPGEGGLTK |
| GLEAN3_25235 | DPGQVTEGVR |
| GLEAN3_25235 | TGTIVTFVK |
| GLEAN3_25235 | VSETVNYACK |
| GLEAN3_25235 | VSETVNYACKDPGQVTEGVR |
| GLEAN3_25926 | SVNEADFVGKPLVIGDR |
| GLEAN3_25927 | LSDIDTIFDTSSFK |
| GLEAN3_25927 | VYELAVTDNK |
| GLEAN3_25966 | CAFPAITTLPK |
| GLEAN3_25966 | ELFLQDNLINSVSR |
| GLEAN3_25966 | GAFSSMTQLQTLR |
| GLEAN3_25966 | IDTSELVCGTPFGVR |
| GLEAN3_25966 | NKIDTSELVCGTPFGVR |
| GLEAN3_25966 | NRPLLNMISNPR |
| GLEAN3_25966 | SPQQTYSLGPGAYR |
| GLEAN3_25966 | TLQLADNPLER |
| GLEAN3_25966 | TVSTGATFILPCTLTAQAAAR |
| GLEAN3_25966 | VFYSLVGDR |
| GLEAN3_25966 | YGGSLCQILKPGNPTK |
| GLEAN3_26000 | CIVILPSGVK |
| GLEAN3_26000 | NQPDTVTVYAGDTIYMR |
| GLEAN3_26000 | SDNTNPQTQYISIR |
| GLEAN3_26000 | VLPADTQPAAVNPR |
| GLEAN3_26000 | VPIPIPTCSMTPPQPSVGQR |
| GLEAN3_26000 | YSDGSIFFTR |
| GLEAN3_26008 | DGKPGPAGAPGEPGNSGPAGASGQR |
| GLEAN3_26008 | GDPGDQGPQGSPGSPGFAGPPGR |
| GLEAN3_26008 | GDQGNPGQPGAQGESGPLGPR |
| GLEAN3_26008 | GEDGGQGSPGAPGLTGEPGK |
| GLEAN3_26008 | GEDGGQGSPGAPGLTGEPGKR |
| GLEAN3_26008 | GEGGSSGPPGPPGPPGPPGPPGQVQSSYGVR |
| GLEAN3_26008 | GEPGQSGSPGQPGLAGTTGPSGER |
| GLEAN3_26008 | GEPGVAGPPGPQGSAGER |
| GLEAN3_26008 | GETGGPGPSGPTGDPGPQGPLGAPGQQGER |
| GLEAN3_26008 | GETGPAGPPGAQGESGER |
| GLEAN3_26008 | GETGSTGAPGPQGPTGAR |
| GLEAN3_26008 | GLPGLVGLPGPQGQR |
| GLEAN3_26008 | GNDGQSGPPGPPGPTGPAGQSGILGLAGGSGPR |
| GLEAN3_26008 | GPGGPAGPPGEAGSR |
| GLEAN3_26008 | GPMGPPGMSGAPGAPGAK |
| GLEAN3_26008 | GPPGPSGSPGPDGPAGAEGDR |
| GLEAN3_26008 | GPQGLTGAQGR |
| GLEAN3_26008 | GSDGSPGPVGAPGPAGPSGQPGER |
| GLEAN3_26008 | GSEGSQGQTGPPGVPGR |
| GLEAN3_26008 | GSTGPAGPSGPSGPAGER |
| GLEAN3_26008 | GSVGPAGPPGGVGER |
| GLEAN3_26008 | RGEPGVAGPPGPQGSAGER |
| GLEAN3_26008 | SGNPGPQGELGPTGAR |
| GLEAN3_26009 | DGPAGPVGLVGGR |
| GLEAN3_26009 | GAAGLQGASGIVGER |
| GLEAN3_26009 | GEAGEGGNMGASGPVGAVGNPGQR |
| GLEAN3_26009 | GGLGVSGDAGEQGLR |
| GLEAN3_26009 | GGVGGLGEMGTQGDR |
| GLEAN3_26009 | GPQGLTGPMGPQGSAGPMGMSGPR |
| GLEAN3_26009 | MGPSGPVGVR |
| GLEAN3_26629 | GASFAGLGR |
| GLEAN3_26629 | TVSQCNDIDECAR |
| GLEAN3_26630 | SAYGGSSQTYFR |
| GLEAN3_26949 | ATDSTLTLETLK |
| GLEAN3_26949 | DSTQMLVDVGTQDTTEK |
| GLEAN3_26949 | EYGLVPIVQETYAQDR |
| GLEAN3_26949 | GANLTPQISCYEETSK |
| GLEAN3_26949 | MFDSQAYSDTDLLFK |
| GLEAN3_26949 | NANEPYYDYAGAFR |
| GLEAN3_26949 | TYLGDYANTIDGLK |
| GLEAN3_26949 | YAGIAVVR |
| GLEAN3_26997 | GPLPDVPEGYANYVAGEQSLYVEPYLK |
| GLEAN3_26997 | GYDLVTSSK |
| GLEAN3_26997 | TNPSPEGQQPSSPGR |
| GLEAN3_27169 | DNIVIGGQAGVYDPNR |
| GLEAN3_27169 | GNFGGNLFWIK |
| GLEAN3_27169 | HQNVAEIINVNSPVELVTAPLIR |
| GLEAN3_27169 | IPDYWVVDNER |
| GLEAN3_27169 | MQAPAFGLASTTFK |
| GLEAN3_27169 | MVIINANIQNAGVYR |
| GLEAN3_27527 | ELISNASDALDK |
| GLEAN3_27527 | GVVDSDDLPLNVSR |
| GLEAN3_27906 | LLDIADFDSFR |
| GLEAN3_27906 | NLYTHTLPFYQPPTGQQLDFIPPEK |
| GLEAN3_27906 | NPGNPNQPVR |
| GLEAN3_28450 | VLSPELYPR |
| GLEAN3_28450 | YDLDITSLR |
| GLEAN3_28748 | ALMAPFYAGYIPDFQLPYDDQQGIQR |
| GLEAN3_28748 | FDELEQR |
| GLEAN3_28748 | FGVDFLGCDPNK |
| GLEAN3_28748 | LPANLDAVISFSEFSK |
| GLEAN3_28748 | TYDAITLIR |
| GLEAN3_28749 | GAPSNIDAIFEKPGGTTVMIK |
| GLEAN3_28749 | GELYTFSGALMWR |
| GLEAN3_28749 | NSQLVQGYPVR |
| GLEAN3_28749 | NVLEDIPGLPLGIDAAFSSK |
| GLEAN3_28749 | TASYFVR |
| GLEAN3_28749 | TTITFTFDNYTPDLPMNQVR |
| GLEAN3_28749 | VWSDVTPLK |
| GLEAN3_28749 | YDHSSGSLSQGFPR |
| GLEAN3_28749 | YVLSGASWGR |
| GLEAN3_28749 | YWEYSGVNLKPGFPR |

The peptides are arranged according to increasing Glean3 entry number of the corresponding proteins. Peptides shared with human proteins are shaded yellow.
